# The P450 *CYP6Z1* confers carbamate/pyrethroid cross‐resistance in a major African malaria vector beside a novel carbamate‐insensitive N485I *acetylcholinesterase‐1* mutation

**DOI:** 10.1111/mec.13673

**Published:** 2016-06-15

**Authors:** Sulaiman S. Ibrahim, Miranda Ndula, Jacob M. Riveron, Helen Irving, Charles S. Wondji

**Affiliations:** ^1^ Vector Biology DepartmentLiverpool School of Tropical MedicinePembroke PlaceLiverpoolL3 5QAUK

**Keywords:** *acetylcholinesterase 1*, *Anopheles funestus*, carbamates, cross‐resistance, CYP6Z1, malaria, pyrethroids

## Abstract

Carbamates are increasingly used for vector control notably in areas with pyrethroid resistance. However, a cross‐resistance between these insecticides in major malaria vectors such as *Anopheles funestus* could severely limit available resistance management options. Unfortunately, the molecular basis of such cross‐resistance remains uncharacterized in *An. funestus*, preventing effective resistance management. Here, using a genomewide transcription profiling, we revealed that metabolic resistance through upregulation of cytochrome P450 genes is driving carbamate resistance. The P450s *CYP6P9a*,*CYP6P9b* and *CYP6Z1* were the most upregulated detoxification genes in the multiple resistant mosquitoes. However, *in silico* docking simulations predicted *CYP6Z1* to metabolize both pyrethroids and carbamates, whereas *CYP6P9a* and *CYP6P9b* were predicted to metabolize only the pyrethroids. Using recombinant enzyme metabolism and inhibition assays, we demonstrated that *CYP6Z1* metabolizes bendiocarb and pyrethroids, whereas *CYP6P9a* and *CYP6P9b* metabolize only the pyrethroids. Other upregulated gene families in resistant mosquitoes included several cuticular protein genes suggesting a possible reduced penetration resistance mechanism. Investigation of the target‐site resistance in *acetylcholinesterase 1* (*ace‐1*) gene detected and established the association between the new N485I mutation and bendiocarb resistance (odds ratio 7.3; *P *< 0.0001). The detection of multiple haplotypes in single mosquitoes after cloning suggested the duplication of *ace‐1*. A TaqMan genotyping of the N485I in nine countries revealed that the mutation is located only in southern Africa with frequency of 10–15% suggesting its recent occurrence. These findings will help in monitoring the spread and evolution of carbamate resistance and improve the design of effective resistance management strategies to control this malaria vector.

## Introduction

Malaria burden remains high in the tropical world with around 584 000 deaths globally in 2013 alone and mostly in African children under the age of 5 (WHO 2014). Malaria control relies largely on the use of insecticides, either as long‐lasting insecticidal nets (LLINs) or as indoor residual spraying (IRS). Pyrethroid insecticides have been the primary choice for most control programmes for the past three decades, but carbamates are increasingly used, either in rotation with pyrethroids or as an IRS replacement for pyrethroids, in areas where pyrethroid resistance is common, such as implemented in Bioko Island (WHO 2012; Hemingway *et al*. 2013). However, resistance to pyrethroids and carbamates is spreading rapidly in *Anopheles* mosquitoes across Africa. In the major vector *Anopheles funestus*, both pyrethroid and carbamate resistances are increasingly reported in southern and West Africa with the fear that such resistance could disrupt malaria control (Brooke *et al*. 2001; Casimiro *et al*. 2006; Cuamba *et al*. 2010; Hunt *et al*. 2010; Djouaka *et al*. 2011; Wondji *et al*. 2012). To date, the molecular basis of carbamate resistance in *An. funestus* remained uncharacterized, whereas significant progress has been made in elucidating the mechanisms of pyrethroid resistance (Wondji *et al*. 2009; Riveron *et al*. 2013; Ibrahim *et al*. 2015). A detailed characterization of the molecular basis of carbamate resistance is a prerequisite for the implementation of suitable resistance management strategies to control this malaria vector.

Previous studies have suggested a possible cross‐resistance between carbamates and pyrethroids in field populations of *An. funestus* (Brooke *et al*. 2001; Wondji *et al*. 2009). Such cross‐resistance could have a devastating impact on malaria control programmes and limit available options for resistance management. Unfortunately, the molecular basis of this cross‐resistance remains uncharacterized and the genes conferring carbamate resistance remain unknown. Elucidation of such cross‐resistance is crucial to facilitate the implementation of successful resistance management strategies and to establish whether methods such as rotation between pyrethroids and carbamates are feasible alternative for resistance management.

Carbamate resistance can also be conferred by a target‐site resistance mechanism with mutations in the *ace‐1* gene which usually induce a resistance to organophosphate insecticides as well (Hemingway & Ranson 2000). Although insensitive acetylcholinesterase was observed in an *An. funestus* population from Mozambique (Cuamba *et al*. 2010), no mutation has so far been detected in this gene from *An. funestus*. In the absence of the common G119S mutation, there was a suggestion that other mutations in this gene could be present and conferring a carbamate‐specific resistance as previously reported in *Drosophila* (Fournier 2005). However, such hypothesis has never been investigated before this study.

To fill these important gaps in knowledge and help improve the management of resistance in field populations of *An. funestus,* we dissected the molecular basis of pyrethroid/carbamate cross‐resistance. Using a microarray‐based genomewide transcription profiling of both carbamate and pyrethroid resistance in *An. funestus* from southern Africa, we detected the genes associated with carbamate resistance and cross‐resistance to pyrethroids. Using *in vitro* recombinant enzyme metabolism and fluorescent probe inhibition assays, we demonstrated that the P450 gene *CYP6Z1* confers cross‐resistance to both insecticide classes, while the main pyrethroid resistance genes *CYP6P9a* and *CYP6P9b* have no metabolic activity towards carbamates. Additionally, cloning of the full length of the *ace‐1* gene detected a new N485I mutation and demonstrated its association with bendiocarb resistance after designing a suitable diagnostic tool. The establishment of this mutation allows detection and tracking of this carbamate resistance marker in the field.

## Materials and methods

### Mosquito collection and rearing

Blood‐fed female *Anopheles funestus* resting indoors were collected in April 2010 and January 2013 between 06:00 and 12:00 a.m. using torches and aspirators from the ceilings and walls of houses in Chikwawa District (0°45′ N, 34°5′E), southern Malawi. This locality has been already described in previous studies (Wondji *et al*. 2012; Riveron *et al*. 2013). The female mosquitoes were forced to lay eggs in 1.5‐mL Eppendorf tubes as described (Morgan *et al*. 2010) and the eggs brought to the Liverpool School of Tropical Medicine (LSTM), where they were reared to adult as previously described (Cuamba *et al*. 2010). The *F*_1_ adults generated from these field‐collected female mosquitoes were randomly mixed in cages for subsequent experiments.

### Insecticide susceptibility bioassays

Insecticide susceptibility assays were performed using 2‐ to 5‐day‐old randomly pooled *F*_1_ adults from Chikwawa as described previously (Cuamba *et al*. 2010; Wondji *et al*. 2012) following the WHO protocol (WHO 1998). Mosquitoes were exposed to 0.1% bendiocarb (carbamate) and to 0.75% permethrin (type I pyrethroid). Synergist assay was also carried out for both insecticides with piperonyl butoxide (PBO). 100 female mosquitoes were pre‐exposed to 4% PBO impregnated paper for 1 h and immediately exposed to the insecticide paper for 1 h. Final mortality was assessed after 24 h.

### Microarray

The 4 × 44k (A‐MEXP‐2245) (Riveron *et al*. 2013) and the 8 × 60k (A‐MEXP‐2374) (Riveron *et al*. 2014a) Agilent microarray chips (Agilent) used in this study contain 60mer probes designed from a set of expressed sequence tags (ESTs) obtained by previous transcriptome sequencing of *An. funestus* (Crawford *et al*. 2010; Gregory *et al*. 2011), in addition to the other gene sequences from *An. funestus* obtained from GenBank (Riveron *et al*. 2013), and also the full transcript set of *Anopheles gambiae*

RNA was extracted from three batches of ten 2‐ to 5‐day‐old *An. funestus* females alive after exposure to 0.1% bendiocarb (resistant, *R*_b_), unexposed F_1_ mosquitoes from Chikwawa to insecticide (control, C) and also from unexposed mosquitoes of the fully susceptible laboratory strain FANG (susceptible, S) using the Picopure RNA Isolation Kit (Arcturus, Foster City, CA, USA). To assess the possible cross‐resistance mechanism, RNA was also extracted from three batches of mosquitoes from Chikwawa alive after exposure to 0.75% permethrin (resistant, *R*_p_). Microarray protocol was performed as previously described (Riveron *et al*. 2013) and data were analysed using genespring gx 12.0 software. More information on the microarray methods is provided in Appendix S1 (Supporting information).

### Quantitative reverse transcriptase PCR (qRT–PCR)

The expression patterns of some of the genes most associated with carbamate or cross‐resistance with pyrethroids from microarray were validated by qRT–PCR using the primers listed in Tables S1–S5 (Supporting information). One microgram of total RNA from three biological replicates for the bendiocarb‐resistant (*R*_b_), permethrin‐resistant (*R*_p_), control (C) (mosquitoes from Chikwawa not exposed to insecticide) and FANG (S) samples was used as the template for cDNA synthesis using Superscript III (Invitrogen) with oligo‐dT20 and RNase H according to the manufacturer's instructions. qRT–PCR amplification was performed as described previously (Kwiatkowska *et al*. 2013; Riveron *et al*. 2013) after normalization with the housekeeping genes ribosomal protein S7 (*RSP7*; AGAP010592) and actin 5C (AGAP000651).

### *In vitro* functional characterization of candidate resistance genes

#### Cloning of the full length of resistance genes

The full length of the candidate resistance genes was amplified from cDNA using the Phusion High‐Fidelity DNA Polymerase (Thermo Scientific) and cloned into the pJET1.2/blunt cloning vector (Thermo Scientific). The primers used are listed in Table S5 (Supporting information). After sequence analysis, one clone that was predominant in the resistant mosquitoes was selected for further analysis.

#### Cloning and heterologous co‐expression of candidate P450s in *Escherichia coli*

The *CYP6Z1* gene was fused to a bacterial ompA+2 leader sequence and expressed in *E. coli JM109* cells using the pCW‐ori+ vector as previously described (Pritchard *et al*. 1998; McLaughlin *et al*. 2008; Stevenson *et al*. 2011). The cloning and expression of *CYP6M7*,* CYP6P9a* and *CYP6P9b* have been previously described (Riveron *et al*. 2014a). The same protocol was used for expression of *CYP6Z1*. Further details of the cloning of the expression construct are provided in the Appendix S1 (Supporting information).

#### Membrane preparation

The recombinant protein of *CYP6Z1* was co‐expressed with *ompA+2* modification together with *An. gambiae CYP450 reductase* in *E. coli JM109* at 21 °C and 150 rpm as described previously (Riveron *et al*. 2014a). Optimal expression was obtained 36–40 h postinduction with 0.5 mm δ‐ALA and 1 mm IPTG. Determination of P450 content, reductase activity and preparation of the ancillary protein cytochrome b_5_ from *An. gambiae* were performed as previously described (Omura & Sato 1964; Stevenson *et al*. 2011).

#### Pyrethroid and carbamate metabolism assays

Pyrethroids and carbamates were dissolved in HPLC grade methanol to give 2 mm stock insecticides and metabolism assays carried out with a final concentration of 20 μm insecticides as described previously (Muller *et al*. 2008; Stevenson *et al*. 2011; Riveron *et al*. 2013). Recombinant CYP6P9a, CYP6P9b and CYP6M7 were tested for activities with pyrethroid and carbamate insecticides. Further details on this are provided in Appendix S1 (Supporting information).

#### Kinetics analysis

Kinetic analysis with pyrethroids and bendiocarb was carried out with conditions established as linear with time and amount of enzyme. Steady‐state parameters were obtained by measuring the rate of reaction under linear conditions for 10 min while varying the substrate concentration from 0 to 20 μm. Reactions were performed in triplicates with +NADPH (experimental tubes) in parallel with –NADPH (negative control). *K*_M_ and Vmax were established from the plot of substrate concentrations against the initial velocities and fitting of the data to the Michaelis–Menten equation or sigmoidal module in the case of bendiocarb, using the nonlinear regression as implemented in the graphpad prism 6.03 (GraphPad Software Inc., La Jolla, CA, USA). Catalytic constants and efficiencies were automatically predicted from the steady‐state parameters by the Prism software.

#### Fluorescent probe assays

In order to determine potential O‐dealkylation properties of recombinant CYP6Z1, CYP6P9a and CYP6P9b assays were conducted with fluorescent probes: resorufin‐based probes (resorufin methyl ether: RME, 7‐ethoxyresorufin: 7‐ER, resorufin benzyl ether: RBE), coumarins (7‐ethoxy‐4‐triflouromethylcoumarin: 7‐EFC, 7‐methoxy‐4‐trifluoromethylcoumarin: 7‐MFC) and diethoxyflourescein (DEF). Initially, 1‐μm probes were screened with 10 pmol P450 membranes in a total volume of 250 μL containing 1 mm glucose‐6‐phosphate (G6P), 0.1 mm NADP^+^ and 1 U/mL glucose‐6‐phosphate dehydrogenase (G6PDH), buffered with 50 mm potassium phosphate buffer (pH 7.4) containing 5 mm MgCl_2_. Protocol was as described previously (McLaughlin *et al*. 2008; Stevenson *et al*. 2012) and further details are provided in Appendix S1 (Supporting information).

#### Inhibition assay

For each enzyme, inhibition assays were conducted with DEF, test inhibitors (permethrin, deltamethrin, bendiocarb and propoxur) and miconazole (a positive P450 inhibitor) to assess the degree of binding to pyrethroids and carbamates in the presence of high affinity probe, DEF. The assay was carried out as described in previous studies (Bambal & Bloomer 2006; Kajbaf *et al*. 2011). Further details are provided in Appendix S1 (Supporting information). Results were analysed as above and IC_50_ values calculated from the residual activities towards DEF in the presence of insecticides.

### Homology modelling of P450s and docking of insecticides

In order to understand the binding free energy and binding conformations of pyrethroid insecticides and bendiocarb in CYP6P9a, CYP6P9b and CYP6Z1, 3D homology models of these P450s were created based on the crystal structure of human CYP3A4 (PDB:1TQN) (Yano *et al*. 2004) using the modeller *9v2* (Sali & Blundell 1993). CYP3A4 has 33% identity for CYP6P9a and 32% identity for both CYP6P9b and CYP6Z1. Virtual data sets of ligand insecticides: *cis*‐permethrin (ZINC01850374), deltamethrin (ZINC01997854) and bendiocarb (ZINC02015426), were retrieved from the library of ZINC^12^ (https://zinc.docking.org/) database in mol2 format (Irwin & Shoichet 2005). Molecular docking was carried out using gold5 *v2* (Jones *et al*. 1997), with ChemScore scoring‐a fitness function that has been trained by regression against binding affinities (Eldridge *et al*. 1997) and an active site radius of 20Å centred on the haem iron. For each insecticide, 50 binding solutions were generated and analysed based on binding parameters (scores) and conformation. Analysis of docking conformations and preparation of figures were carried out using the pymol 1.7 (DeLano 2004).

### Detection of target‐site resistance mutation in the acetylcholinesterase gene

#### Polymorphism analysis of *ace‐1* fragment spanning G119S

A fragment of the acetylcholinesterase gene (*ace‐1*) spanning the G119S and F455W mutations previously associated with carbamate resistance (Weill *et al*. 2003; Nabeshima *et al*. 2004) was initially amplified and sequenced in bendiocarb‐resistant and susceptible mosquitoes from Chikwawa in Malawi in order to detect the presence of these two mutations or others associated with carbamate resistance. The PCR amplification, sequencing and analysis were carried out as previously described (Riveron *et al*. 2013) using the primers listed in Table S5 (Supporting information).

#### Genotyping of the G119 *ace‐1* position using pyrosequencing

To further assess the presence or absence of the common G119S *ace‐1* mutation in *An. funestus*, 100 field mosquitoes from Chikwawa (Malawi) and from Chokwe (Mozambique) were screened using a pyrosequencing assay designed to sequence the region encoding this mutation. PCR amplification and pyrosequencing reactions were carried out as previously described (Wondji *et al*. 2007) according to the manufacturer's instructions using the PSQ 96 SNP Reagent Kit (Biotage AB). The three sequence‐specific primers used for this assays are listed in Table S5 (Supporting information).

### Cloning and sequencing of the full length of the acetylcholinesterase *ace‐1 gene*

The full length of the acetylcholinesterase 1 (*ace‐1*) was amplified using cDNA from bendiocarb‐resistant mosquito samples from Malawi, Mozambique, Benin and from the susceptible FANG strain. Further details of the sequencing procedures are provided in Appendix S1 (Supporting information).

### TaqMan diagnostic assay and assessment of A50T and N485I correlation with bendiocarb resistance

To genotype two of the mutations detected in *ace‐1*, a custom TaqMan assay was designed for each of the mutations. The primer and reporter sequences are provided in Table S5 (Supporting information). The TaqMan reactions were performed in a 10 μL final volume containing 1 × SensiMix (Bioline), 800 nm of each primer and 200 nm of each probe, using the Agilent MX3005P machine. The following cycling conditions were used: 10 min at 95 °C, 40 cycles of 15 s at 92 °C and 1 min at 60 °C. These assays were used to assess the correlation between the genotypes of each mutation and bendiocarb‐resistant phenotypes. Twenty‐five mosquitoes that were dead after 1 h of 0.1% bendiocarb exposure (susceptible) and 25 alive mosquitoes (resistant) from Chikwawa (Malawi, collected in 2013) and Chokwe (Mozambique, 2009) were genotyped. In addition, for the Malawi samples, another comparison was made between the two phenotypes after a 45‐min exposure to bendiocarb.

### Geographical distribution of the *N485I* mutation across Africa

To assess the geographical distribution of the N485I mutation across Africa, 30 field‐collected female *An. funestus s.s*. from nine countries belonging to different regions of Africa were genotyped by TaqMan: Benin (Pahou, collected in 2010–2011), Ghana (Obuasi, 2009), Burkina Faso (Bobo‐Dioualasso, 2010), West Africa; Cameroon (Gounougou, 2011), West‐Central Africa; Uganda (Tororo, 2009) and Kenya (Kisumu, 2012) in East Africa; and Malawi (Chikwawa, 2009), Zambia (Katete District, 2011) and Mozambique (Chokwe, 2009) in southern Africa.

### Polymorphism analysis of the exon 5‐7 spanning N485I mutation and investigation of duplication of *ace‐1* gene

To assess the presence of a selective sweep associated with the N485I mutation, a fragment of the *ace‐1* gene spanning the N485I mutation from exon 5 to exon 7 was amplified and sequenced in ten carbamate‐resistant and ten susceptible mosquitoes from Chikwawa in Malawi. The primers are listed in Table S5 (Supporting information). The PCR conditions and polymorphisms analyses were the same as described above for the fragment spanning G119S.

To assess the possible duplication of the *ace‐1* gene in *An. funestus*, the fragment of the gene spanning exons 5–7 was cloned and sequenced for 5 mosquitoes with different genotypes after TaqMan assay.

### Homology modelling of *An. funestus ace‐1 gene*

To find out whether there is any major difference in the three‐dimensional folding of the wild‐type *ace‐1* compared with the 485I mutant and the spacial positioning of the mutations, homology models were generated with modeller 9*v2* (Sali & Blundell 1993), using the crystal structure, 2C58 of *Torpedo californica* acetylcholinesterase (*Tc*AChE) (Colletier *et al*. 2006) which share 50% identity.

## Results

### Transcription profiling of bendiocarb/pyrethroid resistance using microarray

#### Genes upregulated in bendiocarb‐resistant mosquitoes

Priority was given to the list of probes significantly upregulated in the bendiocarb‐resistant vs. control mosquitoes (*R*_b_‐C) (Tables 1 and S1, Supporting information) as this comparison directly compares mosquitoes with a same genetic background which only differ in the resistance phenotype. In this study, the *R*_b_‐C comparison is likely to detect probes differentially expressed in relation to bendiocarb resistance as the mortality level of 60% provides a contrast of resistance between the alive (R) (100% resistant) and the control wild type with only 40% resistance (60% mortality rate after bendiocarb exposure). In addition, consideration was also given to probes that were commonly upregulated in the R‐S and C‐S (Tables 1 and S1, Supporting information).

**Table 1:** Probes from detoxification genes and genes associated with bendiocarb resistance. Probes upregulated in R‐C comparison with *P* < 0.01 and fold change >2 using 4 × 44k chip. Significant expression in other comparisons for bendiocarb and for permethrin is also indicated

Gene name	*R*_b_‐C	*P* value	*R*_b_‐S	C‐S	*R*_p_‐C	*R*_p_‐S	Description
CYP6P9a	5.2	0.0026	12.6	5.6	7.9	23.2	Cytochrome p450
CYP6P9a	2.4	0.0067	17.3	7.8	3.4	37.1	Cytochrome p450
CD577515.1	3.1	0.0066	4.2	3.9			Cuticle protein
CYP6Z1	7.3	0.0014	2.9		7.8	2.9	Cytochrome p450
EE589616.1	20.0	0.0021	4.7		38.2	9.6	d7‐related 1 protein
EE589737.1	15.5	0.0019	4.9		31.8		d7‐related 1 protein
CYP6Z1	5.0	0.0023	2.5	−2.6	6.1	3.1	Cytochrome p450
EE589504.1	16.0	0.0008	4.6	−9.9	28.4	6.9	d7‐related 1 protein
BU038886	5.9	0.0039	2.5	−3.3	10.3	3.8	Trypsin
EE589855.1	16.7	0.0010	4.3	−12.1	26.1		d7‐related 1 protein
AGAP008292‐RA	6.5	0.0024	2.7	−2.9		4.3	Trypsin
CD578260.1	5.6	0.0014	2.4	−3.7		3.5	Trypsin
combined_c6791 (CYP9J11)	6.8	0.0013	10.8			9.3	Cytochrome p450
CYP6Z1	6.7	0.0021	2.7			2.7	Cytochrome p450
Combined_c6791 (CYP9J11)	5.3	0.0010	10.8			8.6	Cytochrome p450
CYP6P9b	3.1	0.0054	4.2			5.4	Cytochrome p450
CYP6P9b	2.4	0.0058	3.9			4.8	Cytochrome p450
Pseudo_P450_between_6AA2_and_6P9a	3.0	0.0030	2.4				Cytochrome p450
CYP6P4a	2.1	0.0032	3.1				Cytochrome p450
CYP6P5	2.1	0.0065	3.5				Cytochrome p450
Combined_c4812	2.8	0.0062	3.9				Short‐chain dehydrogenase
CD577519.1	3.0	0.0039	3.3				Cuticle protein
CD577517.1	2.9	0.0039	3.0				Cuticle protein
CD577317.1	2.8	0.0023	2.8				Cuticle protein
CD577518.1	2.6	0.0027	2.6				Cuticle protein
CD577694.1	2.5	0.0030	2.9				Cuticle protein
CD664220.1	2.5	0.0014	3.5				Cuticle protein
CD577516.1	2.3	0.0060	3.3				Cuticle protein
CD577316.1	2.2	0.0044	2.8				Cuticle protein
EE589911.1	10.3	0.0014			11.4		GE‐rich salivary gland protein
CD578169.1	8.7	0.0022			12.7		Trypsin
Combined_c8512	5.7	0.0035			4.1		Serine protease 14
Combined_c4956 (CYP4G16)	9.1	0.0022		−9.5	9.6		Cytochrome p450
Combined_c4956 (CYP4G16)	8.7	0.0016		−9.1	10.1		Cytochrome p450

#### Genes overexpressed in all three comparisons

Selection of best possible candidate genes conferring resistance to bendiocarb was done by also considering the probes commonly overexpressed in comparison between resistant and the susceptible FANG strain (*R*_b_‐S) or between the control and FANG (C‐S). The cytochrome P450 gene *CYP6P9a* appears to be the best candidate detoxification gene associated with bendiocarb resistance in the *Anopheles funestus* from Chikwawa, as two out of three probes of this gene were consistently overexpressed in the three *R*_b_‐C, *R*_b_‐S and C‐S comparisons (Table 1). The other gene overexpressed in the 3 comparisons was a cuticle protein gene from the *An. funestus* EST CD577515, suggesting that a reduced penetration through cuticle thickening could be operating beside a detoxification through elevated expression of P450 genes. The transcript CD577515 is 86% identical to the AFUN004204 gene in the newly released *An. funestus* genome which is in turn 92% identical to the cuticle protein gene AGAP003382‐RA in *Anopheles gambiae*.

#### Genes overexpressed in *R*_b_‐C and/or *R*_b_‐S

The list of genes commonly overexpressed in *R*_b_‐C and *R*_b_‐S and likely associated with bendiocarb resistance includes several probes belonging to cytochrome P450 genes. Three probes belonging to the *CYP6Z1* gene are consistently overexpressed in both comparisons with FC of 7.3, 6.7 and 5.0, respectively, in *R*_b_‐C (Table 1). However, one of the *CYP6Z1* probes is downregulated in C‐S, while the two others are not significantly expressed; thus, induction of *CYP6Z1* in relation to bendiocarb resistance could not be ruled out. Two probes belonging to the P450 *CYP9J11* are also overexpressed in both *R*_b_‐C (FC 6.8 and 5.3) and R‐S (FC10.8 and 10.8). This list of cytochrome P450 genes commonly overexpressed in both R‐C and R‐S also includes two probes of the *CYP6P9b* genes, as well as one probe each of *CYP6P4b* and *CYP6P5* (Table 1). Several probes belonging to cuticle protein genes were also overexpressed suggesting the presence of a reduced penetration mechanism against bendiocarb in this *An. funestus* population, and *peritrophin a*, a gene involved in the synthesis of the peritrophic matrix which may be involved in increased excretion of ingested toxic chemicals (increased excretion).

#### Genes overexpressed only in *R*_b_‐C

Analysis of probes only overexpressed in *R*_b_‐C also detected several cytochrome P450 probes, among which two probes belonging to the *CYP4G16* are the most upregulated (FC9.1 and 8.7) (Table 1). This gene is the ortholog of the *CYP4G1* from *Drosophila melanogaster*, a gene established to possess decarbonylase function and involved in cuticular hydrocarbon biosynthesis (Qiu *et al*. 2012). This further suggests a possibility of a reduced penetration resistance mechanism through cuticular thickening in the bendiocarb‐resistant mosquitoes.

#### Probes downregulated

The most downregulated probe in all comparisons (*R*_b_‐S, C‐S and *R*_b_‐C) belongs to several cytochrome oxidase subunit III (FC R‐S ‐120, C‐S ‐51) and also an EST encoding a monkey king protein gene (combined_c1873, AGAP006659) (FC‐20.5 in *R*_b_‐S and ‐46.8 in C‐S) (Table S1, Supporting information). There was also an isoform c gene (AGAP012280) (FC‐31.7 in *R*_b_‐S and 65.2 in C‐S).

### Candidate genes associated with cross‐resistance to bendiocarb and permethrin

Two probes of *CYP6P9a* were the only probes consistently overexpressed in all the 5 comparisons for both the 4 × 44k (Table 1) and the 8 × 60k (Table S2, Supporting information) chips suggesting that *CYP6P9a* overexpression is associated with a cross‐resistance between these two insecticide classes. Probes from *CYP6Z1, CYP6P9b, CYP6M7* and *CYP9J11* were overexpressed in R‐C and R‐S for both insecticides (Tables 1 and S2, Supporting information (Riveron *et al*. 2013)), also suggesting a possible role for these P450 genes in the cross‐resistance. Probes for the salivary D7‐related 1 protein gene and for trypsin were also upregulated in both R‐C and R‐S for bendiocarb and permethrin. The cytochrome P450 *CYP4G16* was the only P450 overexpressed in both R‐C comparisons.

### Validation of candidate genes with quantitative real‐time PCR

**Figure 1:**
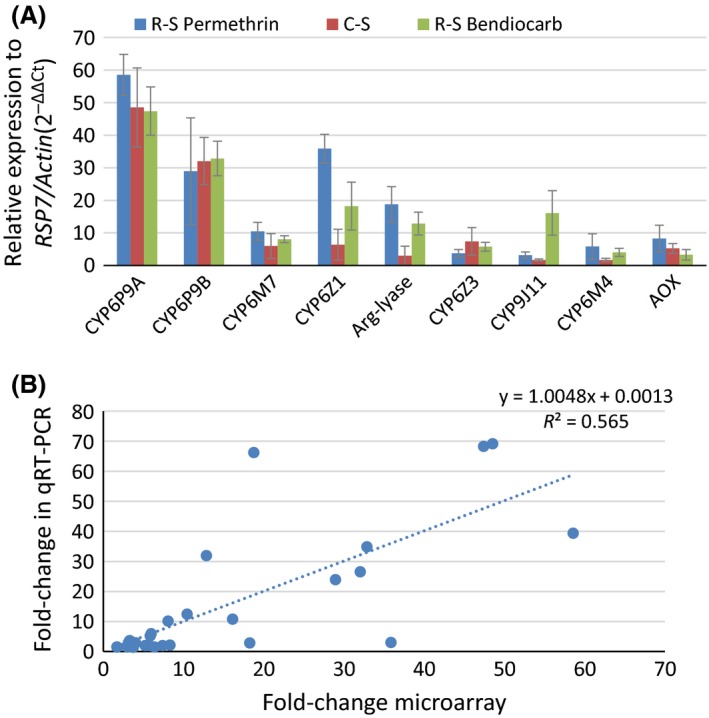
Transcriptional profiling of bendiocarb and permethrin resistance. (A) qRT–PCR differential expression of nine genes (upregulated in microarray) between the susceptible FANG and bendiocarb‐resistant (R‐S bendiocarb), permethrin‐resistant (R‐S permethrin) and control mosquitoes (C‐S). (B) Correlation between microarray data and qRT–PCR data of genes selected from the list of upregulated probes.

The expression profile of the best candidate genes for bendiocarb and cross‐resistance to pyrethroids was similar between the two methods with *CYP6P9a* and *CYP6P9b* fold changes comparable to that seen with microarray (Fig. 1A). Noticeably, the fold change (FC) of these two genes by qRT–PCR was higher han previously obtained in this Malawi populations (Riveron *et al*. 2014b) after further optimization. The other candidate gene *CYP6Z1* was significantly more overexpressed in the permethrin (5.6‐fold)‐ and bendiocarb (2.9‐fold)‐resistant samples than in the control nonexposed samples. This is similar to microarray results suggesting a possible induction of this gene or a higher expression in resistant mosquitoes. Similar trend was observed for the argininosuccinate lyase which was 6‐ and fourfold more overexpressed in permethrin‐ and bendiocarb‐resistant samples, respectively, than the control. The *CYP9J11* was the only gene significantly more overexpressed in resistant bendiocarb than resistant permethrin (sixfold more) and control (ninefold). Overall, there was a significant correlation between qRT–PCR results and microarray (*R* ^2^ = 0.56 when combining all results) (Fig. 1B).

### Validation of the role of *CYP6Z1* in carbamate resistance and cross‐resistance to pyrethroids

In order to confirm whether CYP6P9a, CYP6P9b and CYP6Z1 proteins can metabolize both carbamate and pyrethroid insecticides, recombinant enzymes from these genes were tested using *in vitro* metabolism assays. On average, recombinant CYP6Z1 consistently expressed at a lower concentration (0.10 nmol/mg protein ± 0.05, *n *= 3) than the previously expressed CYP6P9b, CYP6P9a and CYP6M7 (Riveron *et al*. 2013, 2014a) proteins. The enzyme exhibited cytochrome P450 reductase activity of 77.68 nmol cytochrome *c* reduced/min/mg protein ± 5.89.

#### Pyrethroid and carbamate metabolism assays

Activity towards pyrethroid insecticides and relevant kinetic parameters has already been established in the case of CYP6P9a, CYP6P9b and CYP6M7 (Riveron *et al*. 2013, 2014a). Here, we established the metabolic profile of recombinant CYP6Z1 for both type I (permethrin) and type II (deltamethrin) pyrethroids, whereas for the carbamates bendiocarb and propoxur, we assessed the metabolic activity of all the four genes in order to validate which one(s) among them is/are bendiocarb metabolizer(s).

**Figure 2:**
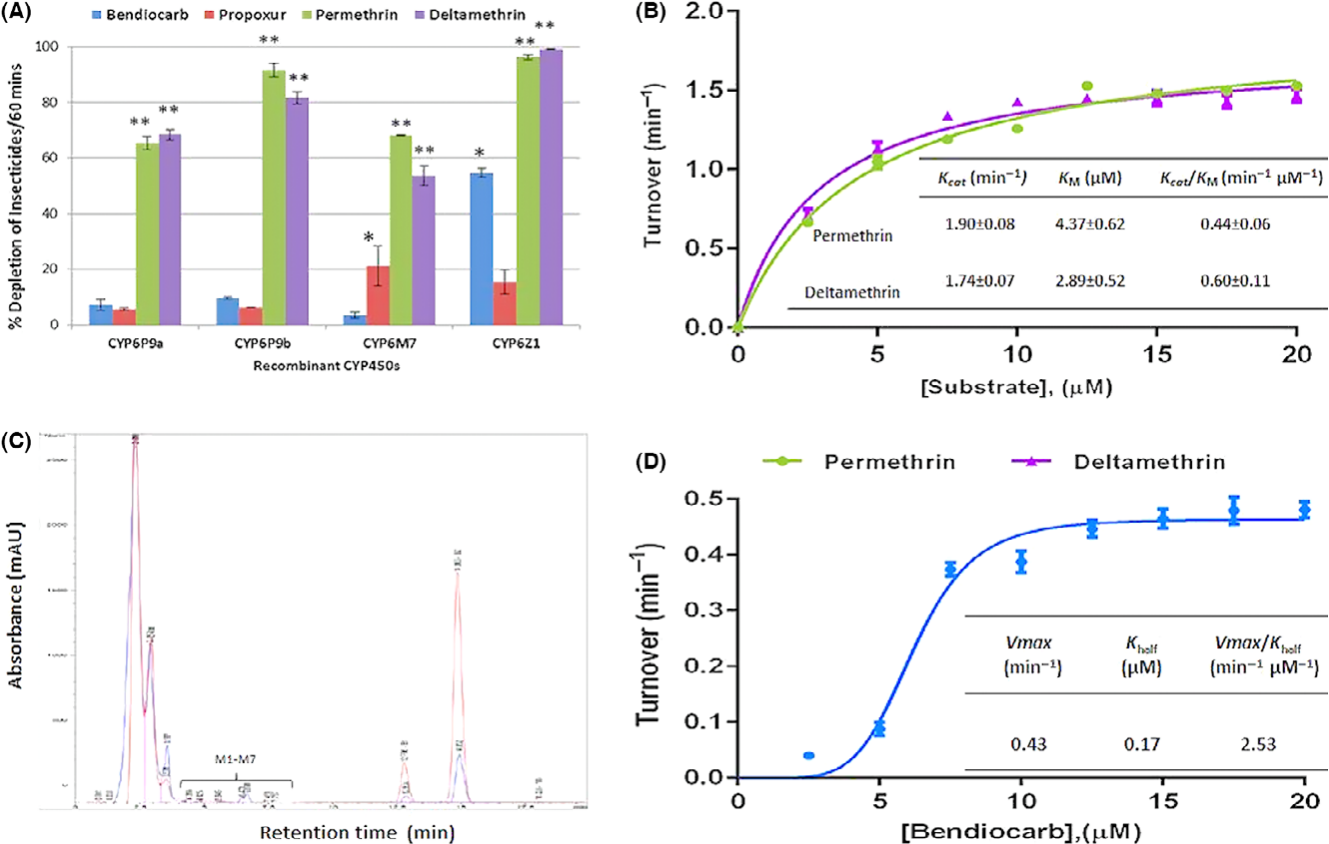
Functional validation of the role of candidate cytochrome P450 genes in carbamate/pyrethroid cross‐resistance. (A) Percentage depletion of 20 μM carbamate and pyrethroid insecticides with *Anopheles funestus* CYP450s. Results are an average of three replicates (*n *= 3) compared with negative control. * and ** Significantly different from negative control (‐NADPH) at *P* < 0.05 BS *P* < 0.01, respectively. (B) Michaelis–Menten plot and kinetic constants of the CYP6Z1‐mediated metabolism of permethrin and deltamethrin. Results are an average of three replicates (*n *= 3) compared with negative control; (C) overlay of HPLC chromatogram of the CYP6Z1 metabolism of bendiocarb with –NADPH in red and +NADPH in blue. Bendiocarb peaks are designated B1 and B2 and putative metabolites peaks from +NADPH samples designated M1‐M7 (4.396–7.75 min) and M8 (18.026 min). (D) Allosteric sigmoidal curve of CYP6Z1 metabolism of bendiocarb. Results are average of three replicates (*n *= 3) compared with negative control. K_half_ = *K*_M_. h = 1.05.

Initial depletion assays revealed that CYP6Z1 metabolizes both permethrin (96.3% depletion after 1 h; *P *< 0.001) and deltamethrin (99.2% depletion; *P* < 0.002) (Fig. 2A). The enzyme exhibited a high affinity towards both permethrin and deltamethrin (Fig. 2B) with *K*_M_ values of 4.4 μm and 2.9 μm, respectively. This is almost threefold lower than values reported for recombinant CYP6P9b (Riveron *et al*. 2014a). CYP6Z1 though has low turnover (low *K*_cat_) for both pyrethroids (1.9/min and 1.7/min, respectively, for permethrin and deltamethrin), resulting in catalytic efficiencies (*K*_cat_/*K*_M_) of 0.43/min/μm and 0.58/min/μm, respectively, for permethrin and deltamethrin. This is comparable to values established for other characterized *An. funestus* P450s (*CYP6P9a, CYP6P9b* and *CYP6M7*) (Riveron *et al*. 2014a).

Metabolism assays with carbamates revealed that CYP6Z1 also metabolizes bendiocarb (54.7% depletion after 1‐h incubation; *P* < 0.01) with possible polar metabolites eluting in the beginning of the HPLC chromatogram (Fig. 2C). However, CYP6Z1 does not show a significant metabolism of propoxur (15.3%; *P* > 0.05) (Fig. 2A). In contrast, CYP6P9a, CYP6P9b and CYP6M7 do not metabolize bendiocarb (<10% depletion after 1‐hr incubation; *P* > 0.05) and propoxur, except for CYP6M7 for which a low but significant depletion was observed (21.2%; *P* < 0.05). Kinetic analysis of CYP6Z1 metabolism of bendiocarb established that this P450 exhibits allosteric kinetics with positive cooperativity (*h* = 1.06) for bendiocarb. Dose–response curve was modelled using the relevant module in the GraphPad Prism, as described (Copeland 2004). The enzyme portrayed sigmoidal curve with high affinity for bendiocarb (low *K*_M_ of 0.17 μm) and low turnover of 0.43/min, suggesting more than one binding site for bendiocarb within its active site. The catalytic efficiency for bendiocarb (*K*_cat_/*K*_half_) was calculated as 2.53/min/μm.

#### Fluorescent probe assay

**Figure 3:**
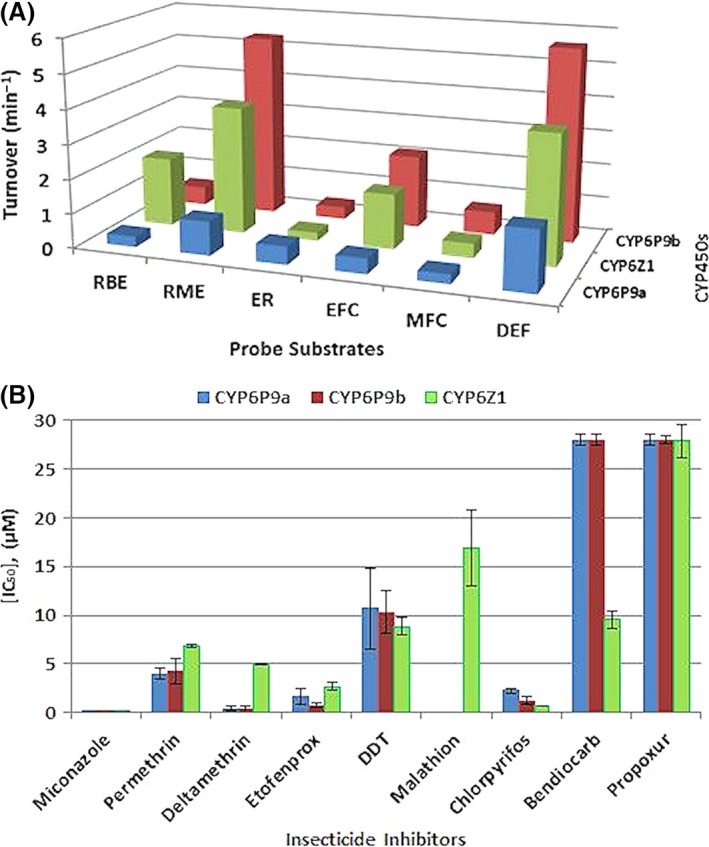
Assessment of biochemical interactions between candidate P450s and different insecticides using fluorescent probes. (A) Testing of O‐dealkylation activities of CYP6P9a, CYP6P9b and CYP6Z1 using fluorescent probes. The solid bars indicate average of significant turnovers of three experimental replicates compared to negative controls (‐NADPH). (B) Inhibition assays: Mean IC_50_ values of the test insecticide inhibitors against CYP450‐mediated dealkylation of DEF. Data represent mean IC_50_ at eight concentrations of each insecticide plus or minus standard deviation. Error bars represent the variation in the values of the IC_50_ between different concentrations.

The potential O‐dealkylation properties of the recombinant CYP6Z1, CYP6P9a and CYP6P9b was investigated with six fluorescent substrates, in order to identify the most suitable probe to conduct inhibition assays. All the three recombinant P450s exhibited preferential activity towards diethoxyfluorescein (DEF), but CYP6P9b and CYP6Z1 portrayed good activity as well with methoxyresorufin (Fig. 3A). Kinetic analysis revealed that CYP6P9a‐, CYP6P9b‐ and CYP6Z1‐mediated dealkylation of DEF followed Michaelis–Menten mechanism (Fig. S2A, Supporting information). Both CYP6P9b and CYP6Z1 portrayed strong affinity towards DEF with *K*_M_ fourfold less than that of CYP6P9a (Table S3, Supporting information). CYP6Z1 has high turnover for diethoxyfluorescein with catalytic efficiency more than 500‐fold than obtained with CYP6P9a and more than sixfold than the efficiency of CYP6P9b (Fig. S2B, Supporting information).

#### Inhibition assays

To further assess the degree of binding of these three enzymes to insecticides, the ability of the various insecticides to inhibit the activity of CYP6Z1, CYP6P9a and CYP6P9b was evaluated in the presence of the DEF substrate. The lowest IC_50_ values (Figs 3B, and S2C, Supporting information) were obtained with miconazole a known potent P450 inhibitor (Lupetti *et al*. 2002). Type II pyrethroid deltamethrin showed the most potent inhibitory activity against the de‐ethylation of DEF, with IC_50_ of <0.5 μm for CYP6P9a and CYP6P9b, indicating strong binding with affinity higher than obtained with type I pyrethroid permethrin (IC_50_ ~4–5 μm). CYP6Z1 shows lower affinity for both permethrin and deltamethrin compared with the other P450s with IC_50_ slightly lower with deltamethrin than permethrin. All three P450s show comparable affinity towards pseudo‐pyrethroid etofenprox and strong affinity towards the organophosphate chlorpyrifos (Fig. 3B). Malathion was only tested with recombinant CYP6Z1 which showed high IC_50_ towards the organophosphate insecticide.

For CYP6P9a and CYP6P9b, very high IC_50_ values (>25 μm) were obtained with carbamates bendiocarb and propoxur indicating that these insecticides are not good binders of these P450s. In contrast, for CYP6Z1, IC_50_ values of 9.5 μm and 8.9 μm, respectively, were obtained for bendiocarb and DDT indicative of good binding.

### Comparative modelling and docking simulation of insecticides

In order to further understand the reasons why CYP6P9a and CYP6P9b could only metabolize pyrethroids while CYP6Z1 metabolizes both pyrethroids and bendiocarb, a 3D homology models of these P450s were created. The CYP6Z1 metabolizes pyrethroids because it accommodates both permethrin and deltamethrin which docked into its active site productively with the 4ʹ spot of benzyl ring above the haem at a distance of 4.7Å and 5.0Å, respectively (Fig. S2D and E, Supporting information). In this posture, ring hydroxylation, characteristic of pyrethroid metabolizers is possible (Stevenson *et al*. 2011). Permethrin and deltamethrin have also been shown to dock within the active site of CYP6P9b productively with trans‐methyl group and 4ʹ spot of the phenoxy ring oriented for hydroxylation (Ibrahim *et al*. 2015).

**Figure 4:**
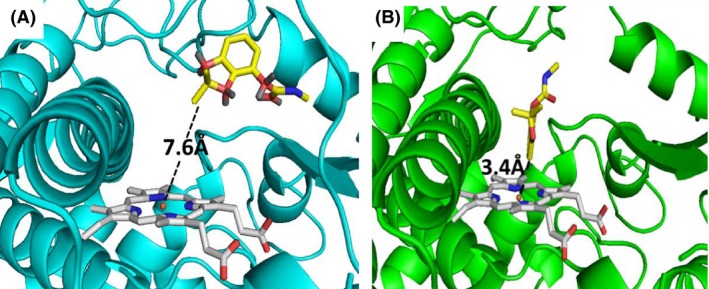
Comparative *in silico* docking of bendiocarb to P450 models. (A) Bendiocarb in the active site of CYP6P9b. Bendiocarb is in stick format and yellow, while CYP6P9b is presented as helices and cyan. Haem atoms are in stick format and grey. Distance between possible sites of metabolism and haem iron is annotated in Angstrom. (B) Conformation of bendiocarb in the active site of CYP6Z1. CYP6Z1 is presented in green helices.

However, with bendiocarb, lower ChemScore and higher free energy of binding (Table S3, Supporting information) were observed when the carbamate docked into the active sites of CYP6P9a and CYP6P9b. This is in contrast with the higher values obtained from docking of bendiocarb with CYP6Z1. The insecticide docked in the active site of CYP6P9b closer than in CYP6P9a, but with the 2ʹ dimethyl group oriented towards the haem at a distance of 7.6Å (Fig. 4A), far away for productive binding and optimal metabolism. Not surprisingly, bendiocarb docked into the active site of CYP6Z1 productively, above the haem with the C‐4 of the phenyl ring located 3.4Å from the haem iron (Fig. 4B). In this posture ring, hydroxylation product (4‐hydroxybendiocarb) is predicted to be the major primary metabolite. The carbamate ester group is positioned opposite, pointed away from the haem catalytic site suggesting that metabolism does not proceed via ester hydrolysis to generate benzodioxol‐4‐ol.

### Target‐site resistance through a modified acetylcholinesterase

#### Sequencing of a fragment of the *ace‐1* gene spanning the 119 codon

A 559‐bp aligned fragment consisting of a portion of exon 3 (from position 1 to 208), intron 3 (from 209 to 287), exon 4 (from 288 to 485) and partial intron 4 (from 486 to 559) was obtained from bendiocarb‐resistant and susceptible mosquitoes from Chikwawa, Malawi. A summary of the polymorphism pattern is presented in Table S4 and Fig. S3A (Supporting information). The G119S mutation which confers carbamate and organophosphate resistance in *An. gambiae* and *Culex quinquefasciatus* (Weill *et al*. 2003) was not detected. However, analysis of the polymorphism pattern detected a marginal difference between bendiocarb susceptible and resistant mosquitoes as a lower genetic diversity was observed for resistant mosquitoes particularly in coding regions with less mutations observed in resistant than in susceptible (4 vs. 9), and a reduced genetic diversity in resistant than in susceptible (0.0042 vs. 0.0101). Furthermore, a significant estimate of D* (Fu and Li) was observed for the test of selection for resistant mosquitoes indicating that the acetylcholinesterase gene could be under selection in carbamate‐resistant mosquitoes. The construction of a maximum‐likelihood phylogenetic tree did not reveal a clear clustering of haplotypes according to the resistance phenotypes (Fig. S3B, Supporting information), but nevertheless revealed that some haplotypes form a separate cluster as shown by the phylogenetic tree. Altogether, the polymorphism pattern of this *ace‐1* fragment suggests that a mutation associated with bendiocarb resistance could be present in this gene.

#### Genotyping of 119 coding position

To confirm the absence of the G119S mutation, a pyrosequencing reaction was performed in 100 field‐collected mosquitoes from Chikwawa. All the pyrogram traces showed only the GGA codon which codes for glycine (Fig. S3C, Supporting information) confirming that the G119S mutation is not present in the *An. funestus* population from Chikwawa.

#### Search for potential resistance mutations by sequencing of full‐length *ace‐1*

A direct sequencing of the full‐length cDNA and clones of *ace‐1* (2208 bp; 736 amino acids) detected a total of 45 substitution sites of which 4 were nonsynonymous inducing an amino acid changes, observed only in Malawi and Mozambique, but not in Benin (Table S4; Fig. S3D, Supporting information). The constructed maximum‐likelihood tree of these haplotypes revealed that they cluster according to their country of origin (Fig. S3E, Supporting information). The common residues of the catalytic triad S200 (S358 in *An. funestus* sequence), E327 (E484) and H440 (H598) and the six cysteine residues which form the three intra‐subunit disulphide bonds are all conserved in *An. funestus* (Fig. S4, Supporting information).

The G119S was confirmed to be absent in *An. funestus* particularly. Three of the four amino acid changes observed are located in the signal peptide region of the *ace* precursor which gets cleaved from the mature protein and consequently are unlikely to impact the activity of this enzyme. With reference to the *An. funestus* sequence, these are the A27T (G to A at position 102 bp) and the A36S (G to T at position 127 bp) both detected in Malawi clones, and the A50T (A to G at position 148 bp) detected in all Mozambican clones. The only mutation in the mature protein is the N485I (AAC to ATC at 1928 bp) located 44 amino acid away from the H440 residue of the catalytic triad. Further analysis on this *ace‐1* sequence is provided in Appendix S1 (Supporting information).

#### Correlation between N485I and bendiocarb resistance

Because of its location near the catalytic triad residue H440, the N485I mutation was further genotyped between bendiocarb‐resistant and susceptible individuals, to establish a possible correlation with resistance using two time points. The TaqMan assay unambiguously detected respective genotypes (Fig. S5A, Supporting information) in 25 susceptible and 25 resistant mosquitoes at the two time points.

**Figure 5:**
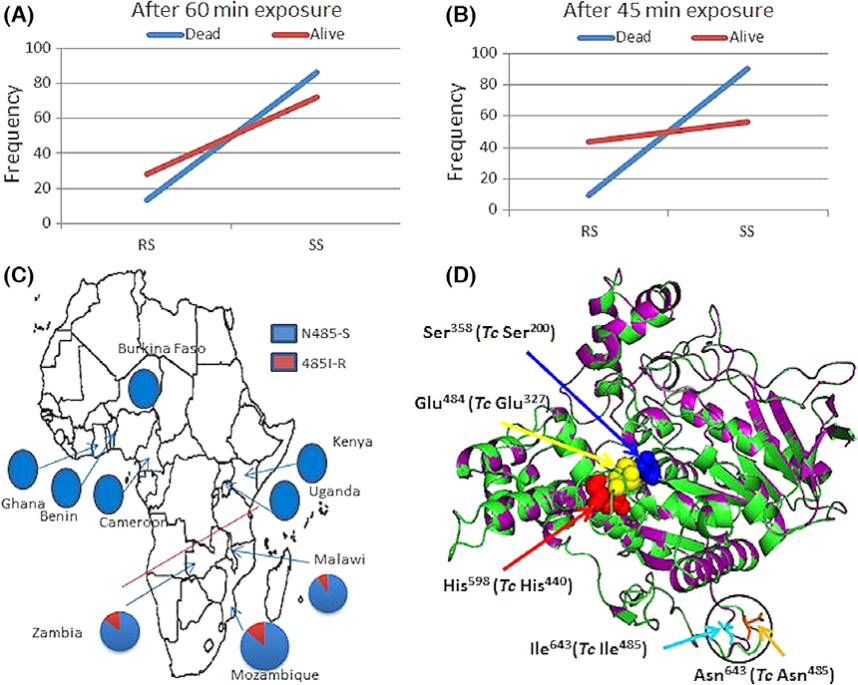
N485I association with bendiocarb resistance across Africa. (A) Evidence of correlation between N485I genotypes and bendiocarb resistance with phenotypes form 60‐min exposure to 0.1% bendiocarb. (B) Evidence of stronger correlation between N485I genotypes and bendiocarb resistance with phenotypes form 45‐min exposure to 0.1% bendiocarb. (C) The N485I geographical distribution across Africa showing a correlation with the bendiocarb resistance distribution in southern Africa. (D) 3D folding of the two generated models of *Anopheles funestus ace‐1* for the wild and mutated alleles at N485I with the template 2C58 revealing a conservative folding. It also reveals that the Asn^485^Ile mutation maps to an external loop considerable distance away from the catalytic hotspot.

At 60‐min exposure time to bendiocarb, a significant correlation was observed between the N485I genotypes (RS and SS) and bendiocarb resistance with an odds ratio (OR) of 2.47 (*P* = 0.012) (Fig. 5A). No homozygote‐resistant genotype (RR) was detected even in resistant mosquitoes (Fig. S5B, Supporting information). When the same analysis was performed with the 45‐min exposure time, a much stronger correlation was observed. Indeed, an OR of 7.3 (*P* < 0.0001) was observed when comparing the frequency of the RS and SS genotypes between phenotypes (Fig. S5C, Supporting information). Furthermore, the correlation was also significant when comparing the frequency of the susceptible and the resistant alleles in both phenotypes with OR = 5.5 (*P* = 0.0004). No homozygote‐resistant genotype (RR) was detected even in resistant mosquitoes (Fig. S5C, Supporting information). In contrast, no such correlation was found for the A50T mutation (data not shown). The correlation of the N485I mutation with bendiocarb resistance indicates that this TaqMan assay is a suitable diagnostic tool to detect and map the distribution of bendiocarb resistance in *An. funestus* in Africa.

#### Geographical distribution of the N485I across Africa

Genotyping 30 mosquitoes in each of ten African countries revealed that the 485I‐resistant allele is only present in southern Africa where bendiocarb resistance is widespread (Fig. 5C). However, the frequency of the 485I‐resistant allele remains relatively low with levels of 10, 14.1 and 15%, respectively, in Malawi, Mozambique and Zambia, all detected as heterozygotes since no homozygote RR mosquito was detected in the three countries. The absence of RR genotype is similar to the case observed in other mosquito species for the G119S mutation which is only detected as heterozygote because of a gene duplication as seen in *An. gambiae* (Djogbenou *et al*. 2009) and *Culex quinquefasciatus* (Labbe *et al*. 2007).

#### Investigation of *ace‐1* duplication in *An. funestus*

To assess the presence of an *ace‐1* duplication in *An. funestus,* a set of 10 bendiocarb‐resistant and 10 susceptible mosquitoes were cloned and sequenced for a 1015‐bp portion spanning N485I from exon 5 to 7. Polymorphism analysis detected evidences for duplication as one resistant mosquito was found to harbour 4 different haplotypes (Fig. S5D, Supporting information) and intron 5 exhibited several sites with multiple polymorphisms. Overall, the *ace‐1* remains polymorphic with no evidence of selection even in resistant mosquitoes (Table S4, Supporting information). However, a phylogenetic tree indicates that all resistant haplotypes harbouring the 485I‐resistant allele cluster tightly (Fig. S5E, Supporting information) suggesting a common origin for this mutation or background selection on such mosquitoes.

### Prediction of structural impact of N485I using homology modelling of *ace‐1*

While most of the mutations which impact on insects acetylcholinesterase activity were reported to map to the location within or adjacent to the active site gorge (Fournier 2005), in contrast the *An. funestus ace‐1* N485I mutation mapped to the external loop joining the α^2^_8,9_ with β9 (Koellner *et al*. 2000), considerable distance away from the catalytic site (Fig. 5D). The only difference observed between the wild‐type and mutant model was in this external loop distorted inward in the mutant model by 3.8Å compared with the wild‐type model and the template, 2C58. Thus, it was difficult to predict the functional relevance of the corresponding outlier residue mutated in the *ace‐1*, through homology modelling alone. Functional characterization using crystal structure in complex with a substrate, *in vitro* and *in vivo* analysis of activities of the wild‐type *An. funestus ace‐1* alongside the mutant, may establish the functional role of this mutant residue. Further analysis on the implication of the spacial positioning of this mutation in the 3D model of the *ace‐1* is provided in the Appendix S1 (Supporting information).

## Discussion

Tackling the growing problem of insecticide resistance is paramount to sustain the progress so far made in reducing the burden of malaria. Understanding the molecular basis of resistance to each insecticide class and possible cross‐resistance pattern is a prerequisite for the implementation of resistance suitable management strategies to control this malaria vector. By deciphering the genetic basis of carbamate resistance in the major vector *Anopheles funestus* and revealing the extent of cross‐resistance with pyrethroids, this study has addressed this important knowledge gap.

### 1‐A cross‐resistance between carbamates and pyrethroids is driven by the cytochrome P450 *CYP6Z1*

This study has revealed a cross‐resistance between carbamates and pyrethroids conferred by cytochrome P450s. Several evidences from this study conclusively point to the major role *CYP6Z1* plays in this cross‐resistance. First, it was consistently among the most overexpressed detoxification genes in resistant mosquitoes. Second, all functional analyses supported the ability of *CYP6Z1* to bind and metabolize bendiocarb. CYP6Z1 exhibited a high affinity to both insecticide classes in contrast to other P450s tested and was able to metabolize bendiocarb with potential polar metabolites which eluted in the beginning of a chromatogram. Indeed, studies have shown that carbamate metabolism proceeds via hydrolysis, oxidative attack and conjugation (Kuhr 1970), and initial reaction has been described to produce very polar products which remain at the origin of the chromatogram, as observed in this study. However, CYP6Z1 was unable to metabolize the other carbamate propoxur. This difference may be due to the presence of benzodioxol ring in bendiocarb and simpler phenyl ring in propoxur, or the presence of isopropoxy group in propoxur and its absence in bendiocarb, differences which could impact on regioselectivity and activity. The cross‐resistance confers by *CYP6Z1* was further supported by the inhibition assays showing that both pyrethroids and carbamates compete to bind the recombinant protein of this gene, as shown by their IC_50_ values. Interestingly, the low IC_50_ towards DDT also suggests that *CYP6Z1* could bind this organochlorine insecticide. *CYP6Z1* involvement in cross‐resistance between carbamate and pyrethroids is in line with a previous study showing that its ortholog in *Anopheles gambiae* also had a broad substrate specificity and could bind productively and metabolize carbaryl and DDT (Chiu *et al*. 2008). Of course, the binding of bendiocarb with its C‐4 of the aromatic ring in close proximity to the haem iron in *An. funestus* CYP6Z1 model is identical to the pattern observed for carbaryl in the active site of *An. gambiae* CYP6Z1, where it binds with its C‐4 and C‐5 close to the haem oxygen (Chiu *et al*. 2008).

Also, cytochrome P450‐mediated cross‐resistance to different insecticides has been previously reported in other resistant insect populations. For example, two P450s, *CYP6P3* and *CYP6M2,* were recently shown to be responsible for cross‐resistance between pyrethroids and carbamates in *An. gambiae* populations from Ivory Coast (Edi *et al*. 2014). Similarly, in *D. melanogaster* cross‐resistance P450s have been detected, such as *CYP6G1* and *CYP12D1* shown to confer cross‐resistance to chemically unrelated insecticides (DDT, nitenpyram and dicyclanil in the case of *CYP6G1*) and DDT and dicyclanil for *CYP12D1* (Daborn *et al*. 2007).

The selection of cross‐resistance genes such as *CYP6Z1* in mosquito populations is a great concern for the continued effectiveness of control strategies as it could limit the options available for insecticide‐based interventions. The presence of broad substrate P450s such as *CYP6Z1* could also confer cross‐resistance to new active ingredients reducing their efficacy before they are even deployed. Therefore, it is imperative to screen the activities of new insecticide active ingredients against multiple resistance enzymes such as *CYP6Z1* in *An. funestus* or *CYP6M2* in *An. gambiae* before deploying them into the field. Further studies will need to establish whether CYP6Z1 confers bendiocarb resistance through an induction or constitutive expression.

However, amidst the danger posed by *CYP6Z1* cross‐resistance, it is good news for resistance management that the highly expressed *CYP6P9a* and *CYP6P9b* genes do not directly metabolize carbamate insecticides. Indeed, despite being also commonly overexpressed in bendiocarb‐ and pyrethroid‐resistant mosquitoes, these two genes were consistently shown by both *in vivo* and *in vitro* functional analyses to be unable to confer carbamate resistance. The inability of the *CYP6P9a* and *CYP6P9b* to directly metabolize carbamates limits the extent of the cross‐resistance since these duplicated genes are by far the major pyrethroid resistance genes in *An. funestus* as shown by the higher overexpression level (20‐ to 30‐fold more overexpressed than *CYP6Z1*) in pyrethroid‐resistant mosquitoes. Therefore, the use of pyrethroids will not automatically select for resistance to carbamates in *An. funestus,* at least not at the same speed as the contributing genes vary also in their relative importance in each resistance. However, there should nevertheless be a note of caution as one cannot rule out the possibility for *CYP6P9a* to be involved in the second stage of biotransformation of the primary bendiocarb product into more water‐soluble metabolites to be conjugated and excreted. Further studies will be needed to assess this hypothesis.

### 2‐Possible role of reduced penetration for bendiocarb resistance in *An. funestus*

This study detected a consistent overexpression of several probes belonging to cuticle protein genes, notably in the *R*_b_‐C comparison. This suggests that besides the upregulation of cytochrome P450 genes, a reduced penetration of insecticides through a thickening of the mosquito cuticle is likely associated with the bendiocarb resistance in *An. funestus*. Furthermore, the overexpression of the *CYP4G16* gene, an ortholog of the *CYP4G1* gene in *D. melanogaster* which is involved in cuticular hydrocarbon biosynthesis (Qiu *et al*. 2012), is an additional support for the presence of a reduced penetration resistance mechanism through cuticle thickening in bendiocarb‐resistant *An. funestus*. However, functional characterization of these putative cuticular genes could establish their actual contribution to the resistance. Cuticle thickening was previously associated with pyrethroid resistance in the *An. funestus ‐*resistant strain FUMOZ‐R originally from Mozambique in southern Africa (Wood *et al*. 2010) further supporting the likely involvement of this mechanism in this species, as also reported in other insect species such as *An. gambiae* (Djouaka *et al*. 2008) and the aphid *Myzus persicae* (Puinean *et al*. 2010).

The overexpression in bendiocarb‐resistant mosquitoes of peritrophin a, a chitin‐binding protein known to strongly bind to peritrophic matrices (Gaines *et al*. 2003), points to potential increased excretion mechanism of resistance in *An. funestus*. The peritrophic matrix has been shown to protect insect by binding toxic chemicals (Lehane 1997) thereby reducing the absorption through the midgut epithelium. This increased excretion has been shown to be the mechanism utilized by the larvae of *Aedes aegypti* which excreted DDT and DDE unchanged (Abedi & Brown 1961).

### 3‐N485I *ace‐1* mutation is associated with bendiocarb resistance

The detection of the N485I mutation in *An. funestus* in southern Africa in this study and its association with bendiocarb resistance shows that carbamate resistance is conferred through multiple resistance mechanisms. Indeed, beside a metabolic resistance through overexpression of P450 and a reduced penetration through cuticular protein genes, this study has demonstrated that target‐site resistance through a modified acetylcholinesterase was also playing an important role in the bendiocarb resistance.

The role of N485I mutation in the bendiocarb resistance was supported by many evidences. First, it was the only amino acid substitution located in the mature protein of *ace‐1* in resistant populations, as the other three mutations are located in the signal peptide which does not impact interaction with substrate (Fournier 2005). Second, the correlation with bendiocarb resistance phenotype was significant and increased markedly when improving the phenotype classification by adjusting the exposure time to the insecticide from 60 to 45 min. Another evidence of the role of the N485I mutation in the bendiocarb resistance is the fact that it is only present in southern Africa where there is widespread carbamate resistance, whereas it is absent in East Africa (Uganda and Kenya) where populations are fully susceptible to bendiocarb (Mulamba *et al*. 2014). However, the absence of the N485I mutation in West Africa where carbamate resistance has been reported (Okoye *et al*. 2008; Djouaka *et al*. 2011) suggests that this mutation plays little or no role in this region. This will be similar to cases in other mosquitoes species such as *Aedes aegypti* and *Aedes albopictus* where both malathion and bendiocarb resistances have been reported but with no involvement of *ace‐1* mutation (Ishak *et al*. 2015). Furthermore, the pattern of distribution of the N485I contrasts completely with that of two other resistance markers in this species: the L119F mutation of the *GSTe2* gene conferring DDT resistance and the A296S of the GABA receptor RDL conferring dieldrin resistance. Indeed, these two markers are predominant in West Africa, but absent in southern Africa (Djouaka *et al*. 2011; Riveron *et al*. 2014a). These differences probably reflect the existence of contemporary barriers to gene flow between *An. funestus* populations across Africa. If such barriers hold, it could limit the spread of the N485I mutation beyond southern Africa. The low frequency of N485I in field populations even among bendiocarb‐resistant populations suggests that this mutation might have been selected recently and may not yet be the predominant resistance mechanism to bendiocarb.

In contrast to the G119S mutation in *An. gambiae* and *Culex quinquefasciatus* (Weill *et al*. 2003, 2004), the N485I does not confer organophosphate resistance. Further functional characterization with the recombinant enzyme from *An. funestus ace‐1* could help to shed further light into the role of N485I on bendiocarb resistance and its lack of activity towards organophosphate insecticides such as malathion. The presence of the new N485I mutation in *An. funestus* and absence of the common G119S is a reminder that what applies in one malaria vector does not necessarily apply to others and that care should be taken in extrapolating findings in one species to all.

The consistent absence of the homozygote RR 485I allele in the three southern African countries suggests a possible fitness cost associated with possessing two copies of the resistant alleles as observed previously for the G119S mutation in *An. gambiae* and *Culex quinquefasciatus* (Djogbenou *et al*. 2010). The evidence of a duplication of *ace‐1* in this study is a further support to such fitness cost as mosquitoes with such duplication are more likely to offset the fitness cost. In contrast to signature of selection observed in *ace‐1* in *An. gambiae* (Edi *et al*. 2014), no such signature is present yet in *An. funestus* and this could be linked to the recent occurrence of the N485I mutation in this species.

## Conclusion

This study has revealed the existence of a P450‐mediated cross‐resistance between pyrethroids and carbamates in the major malaria vector *An. funestus*. Although this is undoubtedly a cause of concern for resistance management, the situation is not yet desperate as this cross‐resistance is only partial because the main pyrethroid resistance genes *CYP6P9a* and *CYP6P9b* are not involved directly, while the N485I mutation is specific to bendiocarb resistance. A resistance management strategies based on a rotation of pyrethroids and carbamates could still be performed, but this should be only be done under close monitoring as there is partial cross‐resistance through *CYP6Z1* **.** The best option recommendable should be the use of organophosphates for control interventions such as IRS despite the challenge of its high cost, since it does not last as long as the pyrethroids or DDT.

## Conflict of interests

The authors declare no competing interests.

C.S.W. conceived and designed the study; S.S.I. performed the biochemical experiments with contribution from J.M.R.; M.N., H.I. and C.S.W. performed the transcriptional analyses; H.I. and C.S.W. performed the experiments and analyses on acetylcholinesterase with contribution from S.S.I.; C.S.W. and S.S.I. wrote the manuscript with contribution from all authors.

## Data accessibility

The DNA sequences reported in this study have been deposited in the GenBank database (Accession nos. JN815117‐JN815138 and KU965594‐KU965669). The microarray data were submitted to Array Express, Accession nos.: E‐MTAB‐3745 (4 × 44k) and E‐MTAB‐3747 (8 × 60k).

## Supporting information


**Fig. S1** Venn diagrams summarising the number of probes differentially regulated in each of the microarray hybridisation (*P* < 0.01 with 2‐fold change).
***Fig. S2** Functional characterisation of candidate resistance genes. *
***Fig. S3** Polymorphism patterns of ace‐1 in field populations of Anopheles funestus.*
***Fig. S4** Alignment of full‐length ace‐1 sequences.*
**Fig. S5** Genotyping of N485I mutation and genetic diversity of the fragment spanning the N485I mutation between bendiocarb susceptible and resistant mosquitoes in Malawi.

**Table S1** Additional probes from detoxification genes and genes associated with bendiocarb resistance.
***Table S2** Probes from detoxification genes or resistance associated genes commonly up‐regulated in both R‐S.*
***Table S3** Kinetic constants for probe substrates and in silico binding parameters of insecticides.*
***Table S4** Genetic parameters for the ace‐1 gene in natural populations of Anopheles funestus.*
**Table S5** List of primers used in this study.

**Appendix S1** Supplementary methods and results.

## References

[mec13673-bib-0001] Abedi Z , Brown A (1961) Peritrophic membrane as vehicle for DDT and DDE excretion in *Aedes aegypti* larvae. Annals of the Entomological Society of America, 54, 539–542.

[mec13673-bib-0002] Bambal RB , Bloomer JC (2006) Screening assay for inhibitors of human cytochrome P‐450. Google Patents.

[mec13673-bib-0003] Brooke BD , Kloke G , Hunt RH *et al* (2001) Bioassay and biochemical analyses of insecticide resistance in southern African Anopheles funestus (Diptera: Culicidae). Bulletin of Entomological Research, 91, 265–272. 11587622 10.1079/ber2001108

[mec13673-bib-0004] Casimiro S , Coleman M , Mohloai P , Hemingway J , Sharp B (2006) Insecticide resistance in *Anopheles funestus* (Diptera: Culicidae) from Mozambique. Journal of Medical Entomology, 43, 267–275. 16619610 10.1603/0022-2585(2006)043[0267:iriafd]2.0.co;2

[mec13673-bib-0005] Chiu TL , Wen Z , Rupasinghe SG , Schuler MA (2008) Comparative molecular modeling of *Anopheles gambiae* CYP6Z1, a mosquito P450 capable of metabolizing DDT. Proceedings of the National Academy of Sciences of the United States of America, 105, 8855–8860. 18577597 10.1073/pnas.0709249105 PMC2449330

[mec13673-bib-0006] Colletier JP , Fournier D , Greenblatt HM *et al* (2006) Structural insights into substrate traffic and inhibition in acetylcholinesterase. The EMBO Journal, 25, 2746–2756. 16763558 10.1038/sj.emboj.7601175 PMC1500847

[mec13673-bib-0007] Copeland RA (2004) Enzymes: A Practical Introduction to Structure, Mechanism, and Data Analysis. John Wiley & Sons, New York, NY, USA.

[mec13673-bib-0008] Crawford JE , Guelbeogo WM , Sanou A *et al* (2010) De novo transcriptome sequencing in *Anopheles funestus* using Illumina RNA‐seq technology. PLoS ONE, 5, e14202. 21151993 10.1371/journal.pone.0014202 PMC2996306

[mec13673-bib-0009] Cuamba N , Morgan JC , Irving H , Steven A , Wondji CS (2010) High level of pyrethroid resistance in an *Anopheles funestus* population of the Chokwe District in Mozambique. PLoS ONE, 5, e11010. 20544036 10.1371/journal.pone.0011010 PMC2882342

[mec13673-bib-0010] Daborn PJ , Lumb C , Boey A , Wong W , Ffrench‐Constant RH , Batterham P (2007) Evaluating the insecticide resistance potential of eight *Drosophila melanogaster* cytochrome P450 genes by transgenic over‐expression. Insect Biochemistry and Molecular Biology, 37, 512–519. 17456446 10.1016/j.ibmb.2007.02.008

[mec13673-bib-0011] DeLano WL (2004) PyMOL User's Guide. The PyMOL Molecular Graphics System

[mec13673-bib-0012] Djogbenou L , Labbe P , Chandre F , Pasteur N , Weill M (2009) Ace‐1 duplication in *Anopheles gambiae*: a challenge for malaria control. Malaria Journal, 8, 70. 19374767 10.1186/1475-2875-8-70 PMC2679766

[mec13673-bib-0013] Djogbenou L , Noel V , Agnew P (2010) Costs of insensitive acetylcholinesterase insecticide resistance for the malaria vector *Anopheles gambiae* homozygous for the G119S mutation. Malaria Journal, 9, 12. 20070891 10.1186/1475-2875-9-12 PMC2816975

[mec13673-bib-0014] Djouaka RF , Bakare AA , Coulibaly ON *et al* (2008) Expression of the cytochrome P450s, CYP6P3 and CYP6M2 are significantly elevated in multiple pyrethroid resistant populations of *Anopheles gambiae* s.s. from Southern Benin and Nigeria. BMC Genomics, 9, 538. 19014539 10.1186/1471-2164-9-538 PMC2588609

[mec13673-bib-0015] Djouaka R , Irving H , Tukur Z , Wondji CS (2011) Exploring mechanisms of multiple insecticide resistance in a population of the malaria vector *Anopheles funestus* in Benin. PLoS ONE, 6, e27760. 22110757 10.1371/journal.pone.0027760 PMC3218031

[mec13673-bib-0016] Edi CV , Djogbenou L , Jenkins AM *et al* (2014) CYP6 P450 enzymes and ACE‐1 duplication produce extreme and multiple insecticide resistance in the malaria mosquito *Anopheles gambiae* . PLoS Genetics, 10, e1004236. 24651294 10.1371/journal.pgen.1004236 PMC3961184

[mec13673-bib-0017] Eldridge MD , Murray CW , Auton TR , Paolini GV , Mee RP (1997) Empirical scoring functions: I. The development of a fast empirical scoring function to estimate the binding affinity of ligands in receptor complexes. Journal of Computer‐aided Molecular Design, 11, 425–445. 9385547 10.1023/a:1007996124545

[mec13673-bib-0018] Fournier D (2005) Mutations of acetylcholinesterase which confer insecticide resistance in insect populations. Chemico‐biological Interactions, 157–158, 257–261. 10.1016/j.cbi.2005.10.040 16274684

[mec13673-bib-0019] Gaines PJ , Walmsley SJ , Wisnewski N (2003) Cloning and characterization of five cDNAs encoding peritrophin‐A domains from the cat flea, *Ctenocephalides felis* . Insect Biochemistry and Molecular Biology, 33, 1061–1073. 14563358 10.1016/s0965-1748(03)00096-1

[mec13673-bib-0020] Gregory R , Darby AC , Irving H *et al* (2011) A de novo expression profiling of *Anopheles funestus*, malaria vector in Africa, using 454 pyrosequencing. PLoS ONE, 6, e17418. 21364769 10.1371/journal.pone.0017418 PMC3045460

[mec13673-bib-0021] Hemingway J , Ranson H (2000) Insecticide resistance in insect vectors of human disease. Annual Review of Entomology, 45, 369–389. 10.1146/annurev.ento.45.1.371 10761582

[mec13673-bib-0022] Hemingway J , Vontas J , Poupardin R *et al* (2013) Country‐level operational implementation of the global plan for insecticide resistance management. Proceedings of the National Academy of Sciences of the United States of America, 110, 9397–9402. 23696658 10.1073/pnas.1307656110 PMC3677419

[mec13673-bib-0023] Hunt R , Edwardes M , Coetzee M (2010) Pyrethroid resistance in southern African *Anopheles funestus* extends to Likoma Island in Lake Malawi. Parasites & Vectors, 3, 122. 21192834 10.1186/1756-3305-3-122 PMC3020165

[mec13673-bib-0024] Ibrahim SS , Riveron JM , Bibby J *et al* (2015) Allelic variation of cytochrome P450s drives resistance to bednet insecticides in a Major Malaria Vector. PLoS Genetics, 11, e1005618. 26517127 10.1371/journal.pgen.1005618 PMC4627800

[mec13673-bib-0026] Irwin JJ , Shoichet BK (2005) ZINC–a free database of commercially available compounds for virtual screening. Journal of Chemical Information and Modeling, 45, 177–182. 15667143 10.1021/ci049714 PMC1360656

[mec13673-bib-0027] Ishak IH , Jaal Z , Ranson H , Wondji CS (2015) Contrasting patterns of insecticide resistance and knockdown resistance (kdr) in the dengue vectors *Aedes aegypti* and *Aedes albopictus* from Malaysia. Parasites & Vectors, 8, 181. 25888775 10.1186/s13071-015-0797-2 PMC4377062

[mec13673-bib-0028] Jones G , Willett P , Glen RC , Leach AR , Taylor R (1997) Development and validation of a genetic algorithm for flexible docking. Journal of Molecular Biology, 267, 727–748. 9126849 10.1006/jmbi.1996.0897

[mec13673-bib-0029] Kajbaf M , Longhi R , Montanari D *et al* (2011) A comparative study of the CYP450 inhibition potential of marketed drugs using two fluorescence based assay platforms routinely used in the pharmaceutical industry. Drug Metabolism Letters, 5, 30–39. 21198439 10.2174/187231211794455262

[mec13673-bib-0030] Koellner G , Kryger G , Millard CB , Silman I , Sussman JL , Steiner T (2000) Active‐site gorge and buried water molecules in crystal structures of acetylcholinesterase from *Torpedo californica* . Journal of Molecular Biology, 296, 713–735. 10669619 10.1006/jmbi.1999.3468

[mec13673-bib-0031] Kuhr RJ (1970) Metabolism of carbamate insecticide chemicals in plants and insects. Journal of Agricultural and Food Chemistry, 18, 1023–1030.

[mec13673-bib-0032] Kwiatkowska RM , Platt N , Poupardin R *et al* (2013) Dissecting the mechanisms responsible for the multiple insecticide resistance phenotype in *Anopheles gambiae* s.s., M form, from Vallee du Kou, Burkina Faso. Gene, 519, 98–106. 23380570 10.1016/j.gene.2013.01.036 PMC3611593

[mec13673-bib-0033] Labbe P , Berthomieu A , Berticat C *et al* (2007) Independent duplications of the acetylcholinesterase gene conferring insecticide resistance in the mosquito *Culex pipiens* . Molecular Biology and Evolution, 24, 1056–1067. 17283366 10.1093/molbev/msm025

[mec13673-bib-0034] Lehane MJ (1997) Peritrophic matrix structure and function. Annual Review of Entomology, 42, 525–550. 10.1146/annurev.ento.42.1.525 15012322

[mec13673-bib-0035] Lupetti A , Danesi R , Campa M , Del Tacca M , Kelly S (2002) Molecular basis of resistance to azole antifungals. Trends in Molecular Medicine, 8, 76–81. 11815273 10.1016/s1471-4914(02)02280-3

[mec13673-bib-0036] McLaughlin LA , Niazi U , Bibby J *et al* (2008) Characterization of inhibitors and substrates of *Anopheles gambiae* CYP6Z2. Insect Molecular Biology, 17, 125–135. 18353102 10.1111/j.1365-2583.2007.00788.x

[mec13673-bib-0037] Morgan JC , Irving H , Okedi LM , Steven A , Wondji CS (2010) Pyrethroid resistance in an *Anopheles funestus* population from Uganda. PLoS ONE, 5, e11872. 20686697 10.1371/journal.pone.0011872 PMC2912372

[mec13673-bib-0038] Mulamba C , Riveron JM , Ibrahim SS *et al* (2014) Widespread pyrethroid and DDT resistance in the Major Malaria Vector *Anopheles funestus* in East Africa Is Driven by metabolic resistance mechanisms. PLoS ONE, 9, e110058. 25333491 10.1371/journal.pone.0110058 PMC4198208

[mec13673-bib-0039] Muller P , Warr E , Stevenson BJ *et al* (2008) Field‐caught permethrin‐resistant *Anopheles gambiae* overexpress CYP6P3, a P450 that metabolises pyrethroids. PLoS Genetics, 4, e1000286. 19043575 10.1371/journal.pgen.1000286 PMC2583951

[mec13673-bib-0040] Nabeshima T , Mori A , Kozaki T *et al* (2004) An amino acid substitution attributable to insecticide‐insensitivity of acetylcholinesterase in a *Japanese encephalitis* vector mosquito, Culex tritaeniorhynchus. Biochemical and Biophysical Research Communications, 313, 794–801. 14697262 10.1016/j.bbrc.2003.11.141

[mec13673-bib-0041] Okoye PN , Brooke BD , Koekemoer LL , Hunt RH , Coetzee M (2008) Characterisation of DDT, pyrethroid and carbamate resistance in *Anopheles funestus* from Obuasi, Ghana. Transactions of the Royal Society of Tropical Medicine and Hygiene, 102, 591–598. 18405930 10.1016/j.trstmh.2008.02.022

[mec13673-bib-0042] Omura T , Sato R (1964) The carbon monoxide‐binding pigment of liver microsomes. I. Evidence for its hemoprotein nature. The Journal of Biological Chemistry, 239, 2370–2378. 14209971

[mec13673-bib-0043] Pritchard MP , Glancey MJ , Blake JA *et al* (1998) Functional co‐expression of CYP2D6 and human NADPH‐cytochrome P450 reductase in *Escherichia coli* . Pharmacogenetics, 8, 33–42. 9511179 10.1097/00008571-199802000-00005

[mec13673-bib-0044] Puinean AM , Foster SP , Oliphant L *et al* (2010) Amplification of a cytochrome P450 gene is associated with resistance to neonicotinoid insecticides in the aphid *Myzus persicae* . PLoS Genetics, 6, e1000999. 20585623 10.1371/journal.pgen.1000999 PMC2891718

[mec13673-bib-0045] Qiu Y , Tittiger C , Wicker‐Thomas C *et al* (2012) An insect‐specific P450 oxidative decarbonylase for cuticular hydrocarbon biosynthesis. Proceedings of the National Academy of Sciences of the United States of America, 109, 14858–14863. 22927409 10.1073/pnas.1208650109 PMC3443174

[mec13673-bib-0046] Riveron JM , Irving H , Ndula M *et al* (2013) Directionally selected cytochrome P450 alleles are driving the spread of pyrethroid resistance in the major malaria vector *Anopheles funestus* . Proceedings of the National Academy of Sciences of the United States of America, 110, 252–257. 23248325 10.1073/pnas.1216705110 PMC3538203

[mec13673-bib-0047] Riveron JM , Ibrahim SS , Chanda E *et al* (2014a) The highly polymorphic CYP6M7 cytochrome P450 gene partners with the directionally selected CYP6P9a and CYP6P9b genes to expand the pyrethroid resistance front in the malaria vector *Anopheles funestus* in Africa. BMC Genomics, 15, 817. 25261072 10.1186/1471-2164-15-817 PMC4192331

[mec13673-bib-0048] Riveron JM , Yunta C , Ibrahim SS *et al* (2014b) A single mutation in the GSTe2 gene allows tracking of metabolically‐based insecticide resistance in a major malaria vector. Genome Biology, 15, R27. 24565444 10.1186/gb-2014-15-2-r27 PMC4054843

[mec13673-bib-0049] Sali A , Blundell TL (1993) Comparative protein modelling by satisfaction of spatial restraints. Journal of Molecular Biology, 234, 779–815. 8254673 10.1006/jmbi.1993.1626

[mec13673-bib-0050] Stevenson BJ , Bibby J , Pignatelli P *et al* (2011) Cytochrome P450 6M2 from the malaria vector *Anopheles gambiae* metabolizes pyrethroids: sequential metabolism of deltamethrin revealed. Insect Biochemistry and Molecular Biology, 41, 492–502. 21324359 10.1016/j.ibmb.2011.02.003

[mec13673-bib-0051] Stevenson BJ , Pignatelli P , Nikou D , Paine MJ (2012) Pinpointing P450s associated with pyrethroid metabolism in the dengue vector, *Aedes aegypti*: developing new tools to combat insecticide resistance. PLoS Neglected Tropical Diseases, 6, e1595. 22479665 10.1371/journal.pntd.0001595 PMC3313934

[mec13673-bib-0052] Weill M , Lutfalla G , Mogensen K *et al* (2003) Comparative genomics: Insecticide resistance in mosquito vectors. Nature, 423, 136–137. 12736674 10.1038/423136b

[mec13673-bib-0053] Weill M , Malcolm C , Chandre F *et al* (2004) The unique mutation in ace‐1 giving high insecticide resistance is easily detectable in mosquito vectors. Insect Molecular Biology, 13, 1–7. 14728661 10.1111/j.1365-2583.2004.00452.x

[mec13673-bib-0054] WHO (1998) Test Procedures for Insecticide Resistance Monitoring in Malaria Vectors, Bio‐efficacy and Persistence of Insecticides on Treated Surfaces: Report of the WHO Informal Consultation, WHO, Geneva, Switzerland, 28‐30 September 1998.

[mec13673-bib-0055] WHO (2012) Global Plan for Insecticide Resistance Management (GPIRM). World Health Organization, Geneva, Switzerland.

[mec13673-bib-0056] WHO (2014) World Malaria Report 2014. World Health Organization, Geneva, Switzerland.

[mec13673-bib-0057] Wondji CS , Morgan JC , Coetzee M *et al* (2007) Mapping a Quantitative Trait Locus conferring pyrethroid resistance in the African malaria vector *Anopheles funestus* . BMC Genomics, 8, 34. 17261170 10.1186/1471-2164-8-34 PMC1790900

[mec13673-bib-0058] Wondji CS , Irving H , Morgan J *et al* (2009) Two duplicated P450 genes are associated with pyrethroid resistance in *Anopheles funestus*, a major malaria vector. Genome Research, 19, 452–459. 19196725 10.1101/gr.087916.108 PMC2661802

[mec13673-bib-0059] Wondji CS , Coleman M , Kleinschmidt I *et al* (2012) Impact of pyrethroid resistance on operational malaria control in Malawi. Proceedings of the National Academy of Sciences of the United States of America, 109, 19063–19070. 23118337 10.1073/pnas.1217229109 PMC3511128

[mec13673-bib-0060] Wood OR , Hanrahan S , Coetzee M , Koekemoer LL , Brooke BD (2010) Cuticle thickening associated with pyrethroid resistance in the major malaria vector *Anopheles funestus* . Parasites & Vectors, 3, 67. 20684757 10.1186/1756-3305-3-67 PMC2924294

[mec13673-bib-0061] Yano JK , Wester MR , Schoch GA , Griffin KJ , Stout CD , Johnson EF (2004) The structure of human microsomal cytochrome P450 3A4 determined by X‐ray crystallography to 2.05‐A resolution. The Journal of Biological Chemistry, 279, 38091–38094. 15258162 10.1074/jbc.C400293200

