# Supplementary material for: The P450 CYP6Z1 confers carbamate/pyrethroid cross‐resistance in a major African malaria vector beside a novel carbamate‐insensitive N485I acetylcholinesterase‐1 mutation
Source: Mol Ecol. 2016 Jun 15;25(14):3436–52. doi: 10.1111/mec.13673 (PMC4950264; doi:10.1111/mec.13673)
Supplement: Supplementary file 1 — Fig. S1 Venn diagrams summarising the number of probes differentially regulated in each of the microarray hybridisation (P < 0.01 with 2‐fold change). Fig. S2 Functional characterisation of candidate resistance genes. Fig. S3 Polymorphism patterns of ace‐1 in field populations of Anopheles funestus. Fig. S4 Alignment of full‐length ace‐1 sequences. Fig. S5 Genotyping of N485I mutation and genetic diversity of the fragment spanning the N485I mutation between bendiocarb susceptible and resistant mosquitoes in Malawi. [file MEC-25-3436-s001.pdf]

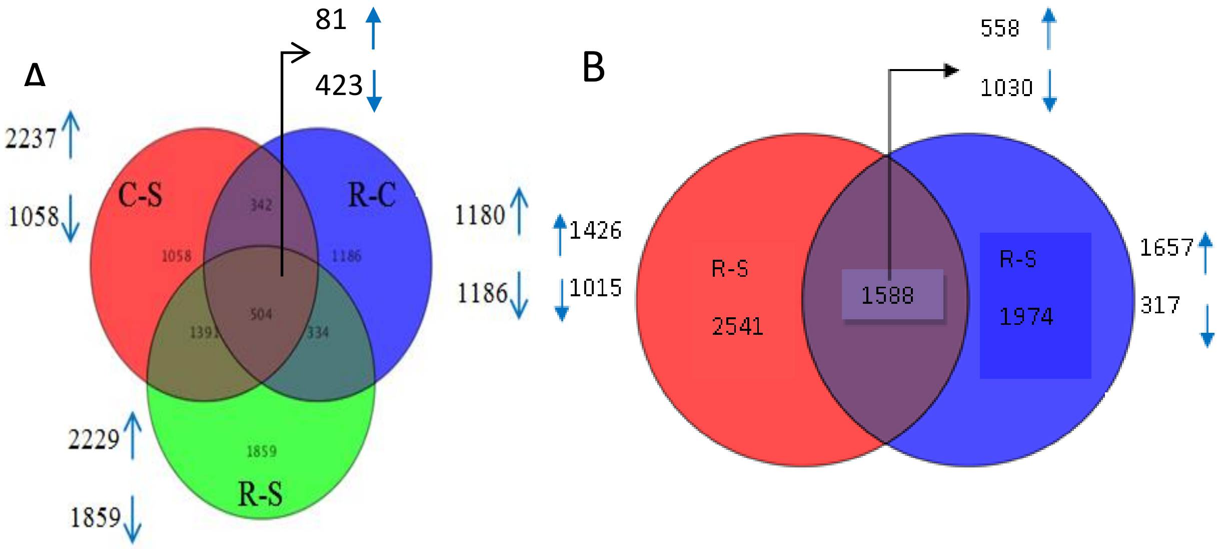

Figure S1: Venn diagrams summarising the number of probes differentially regulated in each of the microarray hybridisation (P<0.01 with 2-fold change). (A) is for bendiocarb resistance analysis with the 4x44k microarray chip, upward arrows indicate up-regulated probes while downward represent down-regulated; (B) is comparison of bendiocarb and permethrin R-S hybridisation with the 8x60k chip.

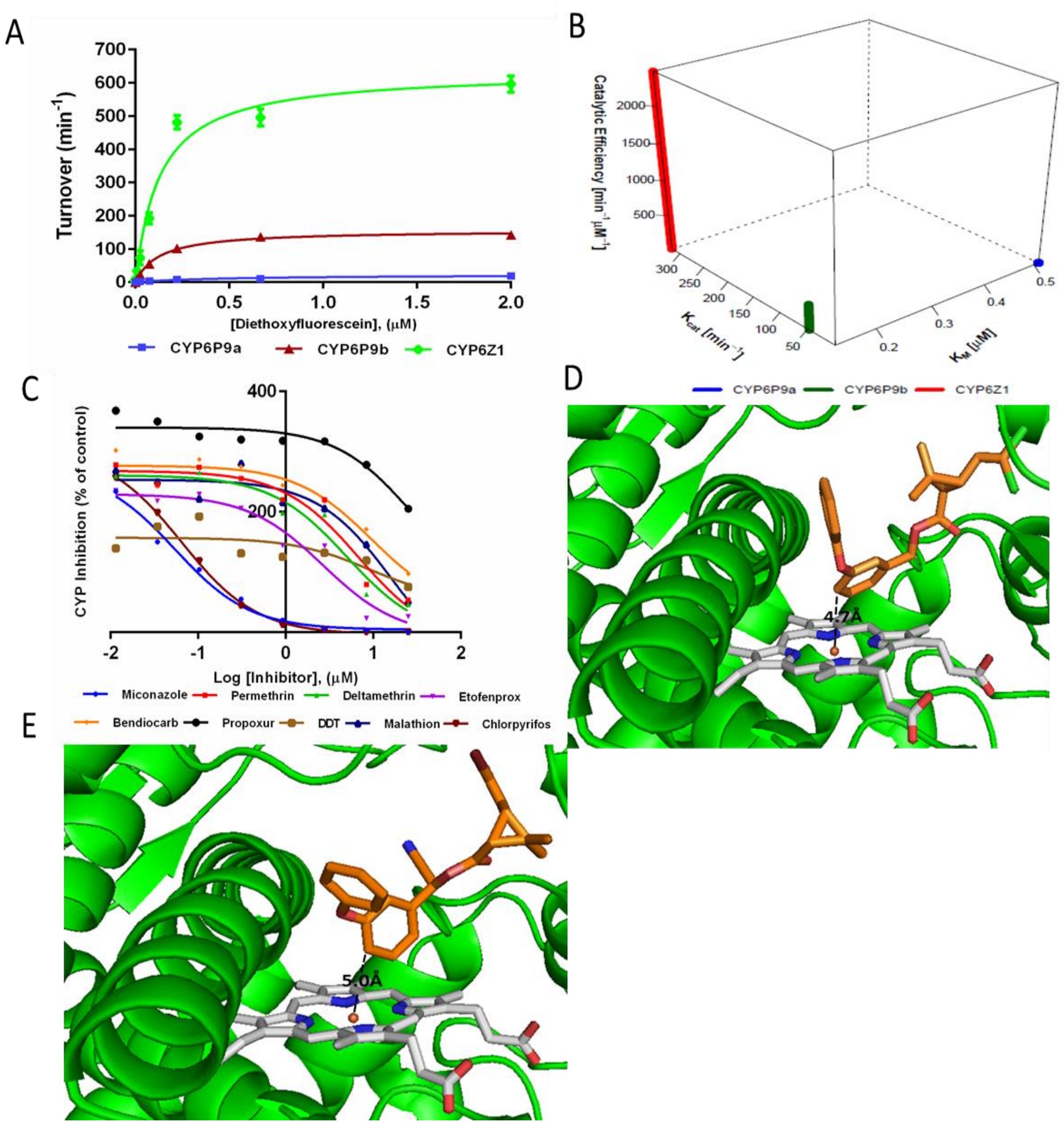

Figure S2: **Functional characterisation of candidate resistance genes:** (A) Michaelis-Menten plot of CYP6P9a, CYP6P9b and CYP6Z1-mediated dealkylation of diethoxyfluorescein. (B) 4D plot of kinetic constants of CYP6P9a, CYP6P9b and CYP6Z1 dealkylation of diethoxyfluorescein; (C) Plot of inhibition of CYP6Z1 metabolism of diethoxyfluorescein by various insecticides. Results are mean ± S.D. of three replicates. Binding conformations of permethrin (D) and deltamethrin (E) in CYP6Z1 model. Permethrin and deltamethrin are in stick format and orange. Heme atoms are in stick format and grey. Distance between possible sites of metabolism and heme iron are annotated in Ängstrom.

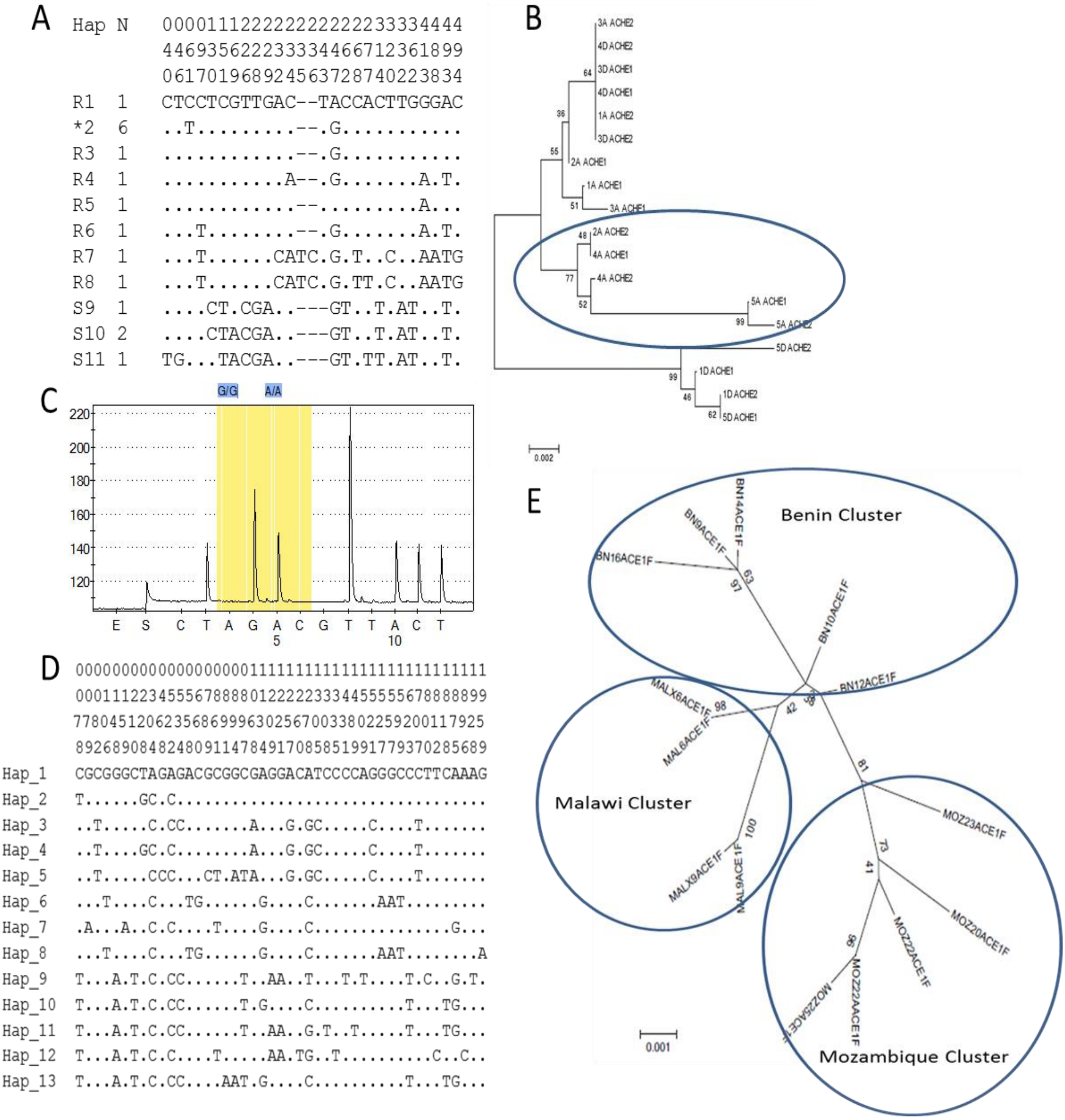

Figure S3: Polymorphism patterns of *ace-1* in field populations of *An. funestus*: (A) Schematic representation of haplotypes of a 559bp portion of the acetylcholinesterase gene in Chikwawa. Only polymorphic sites are shown and these are numbered from the beginning of the 559bp sequence. Dots mean identity with the first sequence while a dash represents an indel. A number has been given to each haplotype preceded by the letter R or S if it is unique to the resistant or susceptible sample respectively. In case of a shared haplotype, the number is preceded by an asterisk. The column (N) indicates the number of individuals sharing the haplotype. (B) Maximum phylogenetic tree of the *ace-1* fragment spanning 119 position. No clear clustering according to phenotypes but nevertheless some resistance haplotypes (Circled) cluster together suggesting possible presence of

a resistance mutation elsewhere in the gene. (C) Pyrosequencing trace of the assay to genotype the GGA codon at 119 position confirming absence of the G119S mutation and showing that for *An. funestus* two mutations are needed (GGA to AGC) to effect substitution. (D) Polymorphic positions in full length sequence of ace-1 across Mozambique (MOZ), Malawi (MAL) and Benin (BN). (E) Maximum likelihood phylogenetic tree of full length ace-1 showing a clustering of haplotypes according to country of origin.

```
Ace-1-Tcalif        ------------------------------------------------------------   0
Ace-1-Cq            MEIRGLITRLLGP--CHLRHLILCSLGLYSILV--QSVHCRHHDIGSSV-----------   45
Ace-1-Fun           MEIRGLLMGRLRLGRRMVPLGLLCITALLLLLPPSAIVQGRHHELNNGAAIGSHQLSG-A   59
Ace-1-gb            MEIRGLLMGRLRLGRRMVPLGLLGVTALLLILPPFALVQGRHHELNNGAAIGSHQLSAAA   60

Ace-1-Tcalif        ------------------------------------------------------------   0
Ace-1-Cq            AHQLGSKYSQSSSLS-------------------SSSQSSSSLAEEATLNKDSDAFFTPY   86
Ace-1-Fun           GAGLSSQSAQSGSLASGVISSAPAAGVSSSS---SLASSGEEDVARITLSKDADAFFTPY   116
Ace-1-gb            GVGLASQSAQSGSLASGVMSSVPAAGASSSSSSSLLSSSAEDDVARITLSKDADAFFTPY   120


Ace-1-Tcalif        ------------------------------------------DDHSELLVNTKSGKVMGT   18
Ace-1-Cq            IGHGDSVRIVDAELGTLEREHIHST-TTRRRGLTRRESSSDATDSDPLVITTDKGKIRGT   145
Ace-1-Fun           IGHGESVRIIDPELGTLEREHVHSGATPRRRGLTRRESNSDANDNDPLVVNTDKGRIRGI   176
Ace-1-gb            IGHGESVRIIDAELGTLE--HVHSGATPRRRGLTRRESNSDANDNDPLVVNTDKGRIRGI   178
                                                              * . *::.*..*:: *
                                                                     [Cys
Ace-1-Tcalif        RVPVLS-SHISAFLGIPFAEPPVGNMRFRRPEPKKPWSGVWNASTYPNNCQQYVDEQFPG   77
Ace-1-Cq            TLEAPSGKKVDAWMGIPYAQPPLGPLRFRHPRPAERWTDR--------------GHVFGD   191
Ace-1-Fun           TVEAPSGKKVDVWLGIPYAQPPVGPLRFRHPRPAEKWNGVLNATTPPNSCVQIVDTVFGD   236
Ace-1-gb            TVDAPSGKKVDVWLGIPYAQPPVGPLRFRHPRPAEKWTGVLNTTTPPNSCVQIVDTVFGD   238
                     :  .  *  .::..::***:*:**:*  :***:*.*  :  *.                 *
                           Cys]
Ace-1-Tcalif        FSGSEMWNPNREMSEDCLYLNIWVPSPRPKSTTVMVWIYGGGFYSGSSTLDVYNGKYLAY   137
Ace-1-Cq            FPGATMWNRNTPLSEDCLYINVFVPRPRPKNAAVMLWIFGGGFYSGTATLDVYDHRTLAS   251
Ace-1-Fun           FPGATMWNPNTPLSEDCLYINVVAPRPRPKNAAVMLWIFGGGFYSGTATLDVYDHRALAS   296
Ace-1-gb            FPGATMWNPNTPLSEDCLYINVVAPRPRPKNAAVMLWIFGGGFYSGTATLDVYDHRALAS   298
                    * *: *** *   :******:*:  .* ****.::**:**:********::*****:  : **

Ace-1-Tcalif        TEEVVLVSLSYRVGAFGFLALHGSQEAPGNVGLLDQRMALQWVHDNIQFFGGDPKTVTIF   197
Ace-1-Cq            EENVIVVSLQYRVASLGFLFL-GTPEAPGNAGLFDQNLALRWVRDNIHRFGGDPSRVTLF   310
Ace-1-Fun           EENVIVVSLQYRVASLGFLFL-GTPEAPGNAGLFDQNLALRWVRDNIHRFGGDPSRVTLF   355
Ace-1-gb            EENVIVVSLQYRVASLGFLFL-GTPEAPGNAGLFDQNLALRWVRDNIHRFGGDPSRVTLF   357
                     *:*::***.***..::*** * *:  *****.**:**.:**:**:***: *****. **:*
                                                                             [Cys
Ace-1-Tcalif        GESAGGASVGMHILSPGSRDLFRRAILQSGSPNCPWASVSVAEGRRRAVELGRNLNCNLN   257
Ace-1-Cq            GESAGAVSVSLHLLSALSRDLFQRAILQSGSPTAPWALVSREEATLRALRLAEAVNCPHD   370
Ace-1-Fun           GESAGAVSVSLHLLSALSRDLFQRAILQSGSPTAPWALVSREEATLRALRLAEAVGCPHE   415
Ace-1-gb            GESAGAVSVSLHLLSALSRDLFQRAILQSGSPTAPWALVSREEATLRALRLAEAVGCPHE   417
                    ** **..**.:*:** ******:*********..*** ** *. **:.*.. : * :
                                Cys]
Ace-1-Tcalif        SD--EELIHCLREKKPQELIDVEWNVLPFDSIFRFSFVPVIDGEFFPTSLESMLNSGNFK   315
Ace-1-Cq            ATKLSDAVECLRTKDPNELVDNEWGTLG---ICEFPFVPVVDGAFLDETPQRSLASGRFK   427
Ace-1-Fun           PSKLSDAVECLRGKDPHVLVNNEWGTLG---ICEFPFVPVVDGAFLDETPQRSLASGRFK   472
Ace-1-gb            PSKLSDAVECLRGKDPHVLVNNEWGTLG---ICEFPFVPVVDGAFLDETPQRSLASGRFK   474
                        .: :.*** *.*. *:: ** .*     * .* ****:** *:  : :  * **.**

Ace-1-Tcalif        KTQILLGVNKDEGSFFLLYGAPGF-SKDSESKISREDFMSGVKLSVPHANDLGLDAVTLQ   374
Ace-1-Cq            KTDILTGSNTEEGYYFIIYYLTELLRKEEGVTVTREEFLQAVRELNPYVNGAARQAIVFE   487
Ace-1-Fun           KTEILTGSNTEEGYYFIIYYLTELLRKEEGVTVSREEFLQAVRELNPYVNGAARQAIVFE   532
Ace-1-gb            KTEILTGSNTEEGYYFIIYYLTELLRKEEGVTVTREEFLQAVRELNPYVNGAARQAIVFE   534
                    **:** * *.:** :*::*      :  *:.  .::**:*:..*:    *:.*  . :*:.::
                                                   [Cys
Ace-1-Tcalif        YTDWMDDNNGIKNRDGLDDIVGDHNVICPLMHFVNKYTKFGNGTYLYFFNHRASNLVWPE   434
Ace-1-Cq            YTDWIEPDNPNSNRDALDKMVGDYHFTCNVNEFAQRYAEEGNNVFMYLYTHRSKGNPWPR   547
Ace-1-Fun           YTDWTEPDNPNSNRDALDKMVGDYHFTCNVNEFAQRYAEEGNNVYMYLYTHRSKGNPWPR   592
Ace-1-gb            YTDWTEPDNPNSNRDALDKMVGDYHFTCNVNEFAQRYAEEGNNVYMYLYTHRSKGNPWPR   594
                    **** : :*  .***.**.:***:.. * : .*.::*:: ** .::*::.**:.    **.

Ace-1-Tcalif        WMGVIHGYEIEFVFGLPLVKELNYTAEEEALSRRIMHYWATFAKTGNPNE--PHSQESKW   492
Ace-1-Cq            WTGVMHGDEINYVFGEPLNSALGYQDDEKDFSRKIMRYWSNFAKTGNPNPSTPSVDLPEW   607
Ace-1-Fun           WTGVMHGDEINYVFGEPLNPSLGYTEDEKDFSRKIMRYWSNFAKTGNPNPNTASSEFPEW   652
Ace-1-gb            WTGVMHGDEINYVFGEPLNPTLGYTEDEKDFSRKIMRYWSNFAKTGNPNPNTASSEFPEW   654
                    * **:** **::*** **     * *   :*: :**:**:**:.********      :  :*
                                                 Cys]
Ace-1-Tcalif        PLFTTKEQKFIDLNTEPMKVHQRLRVQMCVFWNQFLPKLLNATAC---------------   537
Ace-1-Cq            PKHTAHGRHYLELGLNTTFVGRGPRLRQCAFWKKYLPQLVAATSNLQVTPAPSVPCESSS   667
Ace-1-Fun           PKHTAHGRHYLELGLNTSFVGRGPRLRQCAFWKKYLPQLVAATSNIGGEPLPSAPCESSA   712
Ace-1-gb            PKHTAHGRHYLELGLNTSFVGRGPRLRQCAFWKKYLPQLVAATSNLPGPAPPSEPCESSA   714
                    * .*:: :::::*  :   * :  *:: *.**::**:*:: **:

Ace-1-Tcalif        ----------------------      537
Ace-1-Cq            TSYRSTLLLIVTLLLVTRFKI--     688
Ace-1-Fun           FFYRPDLVVLLVSLLTVTVRFIQ     735
Ace-1-gb            FFYRPDLIVLLVSLLTATVRFIQ     737
```

Figure S4: Alignment of full-length *ace-1* sequences. Comparison of full length *An. funestus ace-1* sequence with that of *An. gambiae*, *Cx. quinquefasciatus* and *Torpedo californica*. Residues forming the catalytic triad are highlighted in red and N485 residue in yellow.

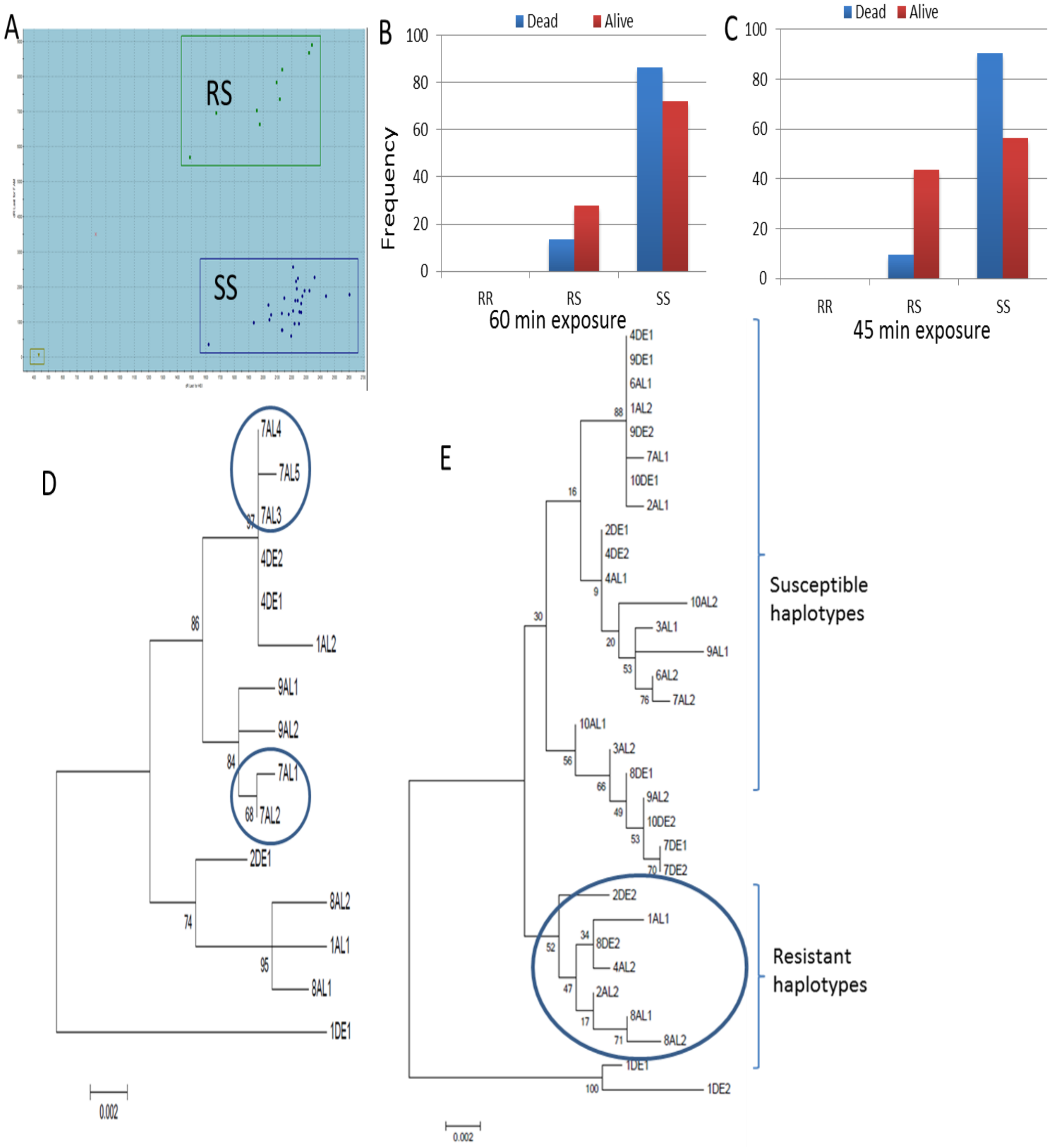

Figure S5: Genotyping of N485I mutation and genetic diversity of the fragment spanning the N485I mutation between bendiocarb susceptible and resistant mosquitoes in Malawi: (A) The results of the TaqMan diagnostic assay for genotyping N485I, with only two genotypes (RS and SS) consistently identified (2 clusters). (B) Genotypes distribution of N485I mutation between resistant and susceptible mosquitoes from Malawi at 60 exposure showing complete absence of homozygotes resistant mosquitoes (RR). (C) is the distribution observed at 45 min. (D) Analysis of *ace-1* duplication: a ML phylogenetic tree of clones of the same fragment of *ace-1* showing an individual

with 4 distinct haplotypes (7AL1, 7AL2, 7AL3/7AL4 and 7AL5) suggesting duplication of this gene. (E) Maximum Likelihood (ML) phylogenetic tree of haplotypes spanning exon5-7 showing that all haplotypes harbouring the 485I mutation (circled) cluster together. AL is for Alive after exposure (resistant mosquitoes) whereas DE is for Dead (susceptible).
