# Supplementary material for: The P450 CYP6Z1 confers carbamate/pyrethroid cross‐resistance in a major African malaria vector beside a novel carbamate‐insensitive N485I acetylcholinesterase‐1 mutation
Source: Mol Ecol. 2016 Jun 15;25(14):3436–52. doi: 10.1111/mec.13673 (PMC4950264; doi:10.1111/mec.13673)
Supplement: Supplementary file 2 — Table S1 Additional probes from detoxification genes and genes associated with bendiocarb resistance. Table S2 Probes from detoxification genes or resistance associated genes commonly up‐regulated in both R‐S. Table S3 Kinetic constants for probe substrates and in silico binding parameters of insecticides. Table S4 Genetic parameters for the ace‐1 gene in natural populations of Anopheles funestus. Table S5 List of primers used in this study. [file MEC-25-3436-s002.pdf]

Table S1: Additional probes from detoxification genes and genes associated with bendiocarb resistance. Probes up-regulated in Rb-C comparison with P<0.01 and Fold-change >2 using 4x44k chip as well as genes down-regulated. Significant expression in other comparisons for bendiocarb and for permethrin are also indicated.

			**Upregulated probes**					
**Probes**	**Gene Name**	**Rb-C**	**P Value**	**Rb-S**	**C-S**	**Rp-C**	**Rp-S**	**Description**
CUST_4508_PI406199772	CD577111.1	7.9	0.0023	-2.7		9.2		atp synthase f0 subunit 6
CUST_356_PI406199772	EE589850.1	3.9	0.0087		-7.1	5.5		d7-related 2 protein
CUST_3440_PI406199772	CD577671.1	6.6	0.0011		-3.6	7.6		peritrophin a
CUST_5118_PI406199772	BU038883	6.1	0.0017		-3.9	7.3		peritrophin a
CUST_4104_PI406201128	AGAP002418-RA	2.8	0.0034					cytochrome p450
CUST_5107_PI406201128	AGAP002204-RA	2.7	0.0030					cytochrome p450 CYP325D1
CUST_157_PI406199798	AGAP000088-RA	2.7	0.0016					cytochrome p450
CUST_16709_PI406201128	AGAP008212-RA	2.5	0.0053					cytochrome p450
CUST_7029_PI406199769	combined_c3556	2.1	0.0093					cytochrome p450
CUST_1946_PI406199798	AGAP002197-RA	2.1	0.0029					cytochrome p450
CUST_369_PI406199788	gb-CYP4H18	2.0	0.0021					cytochrome p450 4d1
CUST_633_PI406199788	gb-GSTO1	3.7	0.0015					glutathione s-transferase
CUST_5640_PI406199769	combined_c2855	2.7	0.0044					short-chain dehydrogenase
CUST_22183_PI406201128	AGAP006226-RA	2.4	0.0022					aldehyde oxidase
CUST_1998_PI406199772	CD664210.1	3.6	0.0064					glutathione peroxidase
CUST_7696_PI406199798	AGAP008141-RA	4.7	0.0042					Argininosuccinate lyase
CUST_15573_PI406201128	AGAP009033-RA	2.4	0.0056					chorion peroxidase
CUST_722_PI406199788	gb-PX5A	2.0	0.0098					oxidase peroxidase
CUST_4047_PI406199772	CD577343.1	2.9	0.0075					cuticle protein
CUST_15404_PI406201128	AGAP006917-RA	2.5	0.0034					gustatory receptor (agap006917-pa)
CUST_8869_PI406199798	AGAP009393-RA	2.4	0.0063					odorant receptor 13a
CUST_1592_PI406199772	EE589544.1	9.2	0.0008					ge rich salivary gland protein
CUST_759_PI406199772	EE589462.1	3.1	0.0022					sg2a salivary protein

CUST_5662_PI406199798	AGAP004581-RA	3.1	0.0078					heat shock protein 70 b2
CUST_3090_PI406199772	CD577854.1	3.1	0.0058					hsp70 binding protein
CUST_17251_PI406201128	AGAP008392-RA	2.4	0.0091					hsp70 binding protein
CUST_14660_PI406199769	combined_c7718	2.3	0.0093					clipb17 protein
CUST_4033_PI406199769	combined_c2039	2.7	0.0066					ctlma3 protein
CUST_924_PI406199798	AGAP000871-RA	2.2	0.0038					ctlma3 protein
CUST_799_PI406199769	combined_c404	3.6	0.0010					serine protease
CUST_15844_PI406199769	combined_c8405	2.5	0.0090					serine protease
CUST_692_PI406199798	AGAP000572-RA	4.8	0.0089					serine protease desc4
CUST_9088_PI406199798	AGAP006416-RA	4.3	0.0088					serine protease sp24d
CUST_5009_PI406199772	BU038937	3.0	0.0065	-3.1	-5.9			cytochrome p450 4g15
CUST_3883_PI406199798	AGAP004247-RA	3.1	0.0056	-2.4	-4.5		2.8	glutathione peroxidase
CUST_4607_PI406199772	CD577060.1	6.4	0.0072	-3.2	-9.9			atp synthase f0 subunit 6
CUST_4583_PI406199772	CD577072.1	6.4	0.0069	-3.3	-9.8			atp synthase f0 subunit 6
CUST_13266_PI406199769	combined_c6787	2.4	0.0093	-2.9	-7.4			clipa6 protein
CUST_5907_PI406199769	combined_c2988	2.9	0.0044		-2.9			Glucosylglucuronosyl transferases
CUST_8727_PI406199769	combined_c4419	2.9	0.0022		-2.7			abc transporter
CUST_2536_PI406199772	CD578133.1	3.8	0.0034		-5.4			oxidase peroxidase
CUST_810_PI406199772	EE589416.1	4.1	0.0018		-9.7			d7-related 2 protein
CUST_1736_PI406199772	EE589383.1	4.6	0.0054		-13.9			d7-related 3 protein
CUST_1719_PI406199772	EE589407.1	12.6	0.0009		-30.2			ge rich salivary gland protein
CUST_923_PI406199772	EE589310.1	3.3	0.0078		-8.1			gsg7 salivary protein
CUST_5373_PI406199798	AGAP004582-RA	3.6	0.0023		-2.6			heat shock protein 70 b2
CUST_3256_PI406199772	CD577765.1	7.3	0.0015		-5.1			lethal essential for life l2efl
CUST_5095_PI406199772	BU038894	6.1	0.0017		-6.8			lethal essential for life l2efl
CUST_5096_PI406199772	BU038894	5.9	0.0018		-6.3			lethal essential for life l2efl
CUST_3255_PI406199772	CD577765.1	4.4	0.0066		-5.7			lethal essential for life l2efl
CUST_3438_PI406199772	CD577672.1	2.8	0.0035		-4.9			peritrophin a

CUST_5673_PI406199769	combined_c2871	3.9	0.0064		-4.9			tep1 protein
CUST_6151_PI406199769	combined_c3111	2.3	0.0053		-4.3			clipb5 protein
CUST_7044_PI406199769	combined_c3564	3.0	0.0063		-4.3			serine protease
CUST_1331_PI406199769	combined_c674	5.1	0.0062		-3.1			serine protease 14
CUST_6793_PI406199769	combined_c3435	2.8	0.0024		-2.7			Trypsin

Down-regulated probes

Probes	Gene Name	Rb-C	P Value	Rb-S	C-S	Rp-S	Rp-C	Description
CUST_18430_PI406201128	AGAP012528-RA	-10.14	0.0081	-4.73	-1.07	-1.46	-4.16	anopheles gambiae pest agap012528-pa
CUST_24436_PI406201128	AGAP012528-RA	-10.1	0.0048	-5.6	-7.0		-10.1	zinc finger ccch domain-containing protein 15-like protein
CUST_9504_PI406199798	AGAP008634-RA	-9.6	0.0074				-9.1	candidate odorant receptor
CUST_909_PI406199798	AGAP001012-RA	-7.4	0.0080	15.5				sec61 protein translocation complex beta subunit
CUST_113_PI406199788	gb-COEAE1D	-6	0.0058	4.9				Carboxylesterase
CUST_2754_PI406201128	AGAP000818-RA CYP9K1	-5	0.0063	9.5				cytochrome P450
CUST_217_PI406199798	AGAP000313-RA	-5.8	0.0093	-2.2	-0.02	-0.05	-2.31	alanine-glyoxylate aminotransferase
CUST_650_PI406199769	combined_c327	-5.5	0.0063	0.42	2.36	-0.42	-2.87	sorbitol dehydrogenase
CUST_9986_PI406199798	AGAP006207-RA	-5.2	0.0053	-0.76	-0.17	-0.21	-0.96	carboxypeptidase b
CUST_11440_PI406201128	AGAP004928-RA	-4.92	0.0055	-2.19	-0.25	-0.18	-2.1	litaf-like protein

Table S2: Probes from detoxification genes or resistance associated genes commonly up-regulated in both R-S. Comparison for bendiocarb and permethrin resistance with the new 8x60k *An. funestus* chip.

Probe Name	Systematic name	FC Rb-S Bendiocarb	P value	FC Rp-S Permethrin	P value	Description
CUST_493_PI426302897	Afun000493	3.7	0.0232	2.2	0.0137	aldehyde oxidase
CUST_7646_PI426302897	Afun007646	2.7	0.0136	2.0	0.0048	aldehyde oxidase
CUST_9227_PI426302897	Afun009227	31.9	0.0160	66.3	0.0046	Argininosuccinate lyase
CUST_7008_PI426302897	Afun007008	3.7	0.0487	2.2	0.0289	ABC transporter
CUST_27_PI406199775	CYP6P9a	24.4	0.0472	39.4	0.0034	cytochrome p450
CUST_30_PI406199775	CYP6P9b	17.8	0.0373	24.0	0.0034	cytochrome p450
CUST_7_PI426302915	CYP6M7	10.2	0.0224	10.1	0.0054	cytochrome p450
CUST_26_PI406199775	CYP6P9a	8.0	0.0444	9.8	0.0039	cytochrome p450
CUST_8_PI426302915	CYP6M7	4.5	0.0052	3.7	0.0031	cytochrome p450
CUST_7861_PI426302897	Afun007861 (CYP6Z1)	3.0	0.0424	3.1	0.0034	cytochrome p450
CUST_7469_PI426302897	Afun007469 (CYP9J5)	3.6	0.0399	3.1	0.0062	cytochrome p450
CUST_25_PI426302915	CYP6Y2	6.1	0.0288	2.9	0.0209	cytochrome p450
CUST_12197_PI426302897	Afun012197 (CYP9J11)	4.4	0.0170	2.8	0.0071	cytochrome p450
CUST_26_PI426302915	CYP6Y2	6.0	0.0329	2.8	0.0097	cytochrome p450
CUST_27_PI426302915	CYP6Z1	3.0	0.0289	2.5	0.0083	cytochrome p450
CUST_29_PI406199775	CYP6P9b	2.4	0.0433	2.5	0.0136	cytochrome p450
CUST_28_PI426302915	CYP6Z1	3.2	0.0208	2.5	0.0087	cytochrome p450
CUST_7369_PI426302897	Afun007369 (CYP6P9a)	4.8	0.0165	2.5	0.0136	cytochrome p450
CUST_3394_PI426302897	Afun003394 (CYP315A1)	4.8	0.0420	2.4	0.0326	cytochrome p450
CUST_9584_PI426302897	Afun009584 (CYP6N4)	4.7	0.0497	3.2	0.0083	cytochrome p450
CUST_7769_PI426302897	Afun007769 (CYP9K1)	3.5	0.0442	2.4	0.0079	cytochrome p450

CUST_899_PI406199772	EE589329.1	3.1	0.0458	7.6	0.0133	d7-related 1 protein
CUST_360_PI406199772	EE589855.1	2.0	0.0368	2.7	0.0259	d7-related 1 protein
CUST_7499_PI426302897	Afun007499	2.3	0.0092	2.9	0.0141	Glutathione transferase
CUST_8241_PI406199769	combined_c4173	3.8	0.0334	4.8	0.0057	glycoprotein 93
CUST_8240_PI406199769	combined_c4173	3.7	0.0450	4.6	0.0054	glycoprotein 93
CUST_1458_PI406199769	combined_c738	3.7	0.0245	3.8	0.0160	short Chain dehydrogenase
CUST_1096_PI406199769	combined_c557	8.9	0.0352	11.8	0.0044	Trypsin
CUST_1097_PI406199769	combined_c557	7.1	0.0433	6.4	0.0097	Trypsin
CUST_9482_PI406199798	AGAP008292-RA	4.0	0.0135	3.9	0.0102	Trypsin
CUST_5111_PI406199772	BU038886	3.2	0.0358	3.7	0.0091	Trypsin
CUST_2287_PI406199772	CD578260.1	3.0	0.0434	3.6	0.0107	Trypsin
CUST_19_PI426302897	Afun000019	3.1	0.0433	3.5	0.0118	Trypsin

Table S3: Kinetic constants for probe substrates and *in silico* binding parameters of insecticides. (A) Kinetic constants for recombinant CYP6P9a, CYP6P9b and CYP6Z1 metabolism of diethoxyfluorescein (values are mean ±S.D. of three independent replicates); (B) binding parameters of the productive poses of permethrin, deltamethrin and bendiocarb in the active sites of CYP6P9a, CYP6P9b and CYP6Z1.

Kinetic Constants				
**Recombinant proteins**	**Amount (pmol)**	**K_{cat} (min^{-1})**	**K_M (μM)**	**K_{cat}/K_M (min^{-1} μM^{-1})**
**CYP6P9a**	10	2.28±0.22	0.51±0.13	4.47±1.25
**CYP6P9b**	3.33	46.93±0.99	0.13±0.01	366.64±29.67
**CYP6Z1**	2.0	317.3±19.30	0.13±0.03	2469.26±577.15

Binding parameters of the productive poses							
**Allele**	**Pose Rank**	**ChemScore (kJ/mol)**	**ΔG (kJ/mol)**	**S(hbond)**	**S(metal)**	**S(lipo)**	**ΔE(clash)**
**Permethrin**							
**CYP6P9a**	2nd	40.72	-43.49	0.00	0.00	353.63	0.15
**CYP6P9b**	2nd	32.39	-32.90	1.76	0.00	215.96	0.04
**CYP6Z1**	2nd	39.19	-41.12	1.00	0.00	307.87	0.22
**Deltamethrin**							
**CYP6P9a**	2nd	38.27	-39.88	0.90	0.00	304.30	0.1
**CYP6P9b**	3rd	31.72	-32.13	1.00	0.00	235.11	0.05
**CYP6Z1**	3rd	36.14	-37.15	0.97	0.00	278.77	0.01
**Bendiocarb**							
**CYP6P9a**	1st	21.02	-21.19	0.99	0.00	135.48	0.00
**CYP6P9b**	1st	21.74	-22.04	1.81	0.00	119.45	0.07
**CYP6Z1**	1st	24.69	-29.32	1.93	0.00	135.46	0.07

Table S4: Genetic parameters for the *ace-1* gene in natural populations of *An. funestus*

	Total region (559 bp)						Coding region (405 bp)					Non-coding region (154 bp)				
**Samples**	**n**	**S**	**H**	**πx10[2]**	**D**	**D***	**S**	**h**	**πx10[3]**	**D**	**D***	**S**	**h**	**πx10[3]**	**D**	**D***
**Susceptible**	8	15	4	1.35	1.54^{ns}	0.85^{ns}	9	4	1.01	1.35^{ns}	0.73^{ns}	6	3	2.07	1.6^{ns}	0.87^{ns}
**Resistant**	10	12	8	0.85	0.57^{ns}	1.17^{*}	4	5	0.42	0.8^{ns}	1.23^{ns}	8	6	2.03	0.36^{ns}	0.96^{ns}
**Total**	18	24	11	1.3	0.2^{ns}	1.17*	12	8	0.91	0.24^{ns}	0.67^{ns}	12	8	2.4	0.13^{ns}	1.45*

	**N**	**S**	**H**	**π**	**Syn**	**NonSyn**	**D**	**D***
***ace-1* full-length**								
Benin	5	17	5	0.00385	17	0	0.21	0.21
Malawi	4	12	3	0.0035	10	2	1.98	1.98
Mozambique	5	22	5	0.00458	21	1	-0.69	-0.56
Total	14	45	13	0.00621	41	4	-0.36	-0.008
***ace-1* Ex5-7 fragment**								
Resistant	18	27	17	0.0038	12	4	-0.46^{ns}	0.66^{ns}
Susceptible	14	34	9	0.0035	18	2	-0.20^{ns}	0.59^{ns}
Total	32	45	23	0.0092	21	5	-0.90^{ns}	-0.60^{ns}

Table S5: List of primers used in this study

	Forward primer	Reverse primer	Expected size (bp)
**Primers used for qRT-PCR**			
CYP6P9a	CAGCGCGTACACCAGATTGTGTAA	TCACAATTTTTCCACCTTCAAGTAATTACCCGC	92
CYP6P9b	CAGCGCGTACACCAGATTGTGTAA	TTACACCTTTTCTACCTTCAAGTAATTACCCGC	97
CYP9J11	CAAATTTAAAGAGTGCGCTAGG	GTAGATGGTGCCAAGGATGG	115
CYP6Z1	GGATTTCCGATGAGGATTGA	GCAGCGTACTTGATTTACGG	78
CYP6M4	CACTATTCTCTCGCCGAAGG	CAAAGGATCCGCCATTCTAC	119
Arg-Lyase	ATTCGTCGATGGGCATGTA	CACTTTTCACGGATCCTTTTG	120
Ald Oxi	GACTGGCAGACGATTGGATT	TGTAATCCAGCAACGGTGTC	134
CYP6Z3	TTTACCCATGCGGATAGAGC	TGGGTTTCCTTTGTACTACACATC	75
CYP6M7	ACGACGGTACGCTAACGACT	TAACGCCAGCTCATACAACG	113
RSP7 *(AGAP010592)*	GTGTTCGGTTCCAAGGTGAT	TCCGAGTTCATTTCCAGCTC	98
Actin *(AGAP000651)*	TTAAACCCAAAAGCCAATCG	ACCGGATGCATACAGTGACA	111
**Primers used for functional characterisation**			
CYP6P9afull	ATGGAGCTCATTAACGTGGTGTTGGC	TCA CAA TTT TTC CAC CTT CAA GTA ATT ACC CGC	
CYP6P9bfull	ATGGAGCTCATTAACGTGGTGTTGGC	TTA CAC CTT TTC TAC CTT CAA GTA ATT ACC CGC	
CYP6Z1full	ATGATCCTTTACGCTATCGCG	TCACACTCTTCTTTCAATCCTC	
OMPA+2 FORWARD	GGAATTCCATATGAAAAAGACAGCTATCGCG		
OMPA+2 CYP6P9a/bF	CAACACCACGTTAATGAGCTCCATCGGAGCGGCCTGCGCTAC GGTAGCGAA		
OMPA+2 CYP6M7F	CAAAATGTCTAGCGGCTCCATCGGAGCGGCCTGCGCTACGGT AGCGAA		
OMPA+2 CYP6Z1F	CACCGCGATAGC GTAAAGGATCAT CGGAGCGGCCTGCGCTACGGTAGCGAA		
OMPACYP6P9aR		TCTAGAGAATTCTCACAATTTTTCCACCTTCAAG	
OMPACYP6P9bR		TCTAGAGAATTCTTACACCTTTTCTACCTTCAAG	
OMPACYP6M7R		TCTAGAGAATTCTCATGTGCTCAGCTTTTCCACC	
OMPACYP6Z1R		TCTAGAGAATTCTCACACTCTTCTTTCAATCCTC	
**G119S *ace-1* pyrosequencing genotyping**			
Forward primer		CCTGTCCGAGGACTGTCTGT	
Reverse primer		ACCACGATCACGTTCTCCTC	
Sequencing		TGTGGATCTTCGGCGG	
Sequence to analyse		5'-C A/G GCTTCTACTCC-3'	
Dispensation order for sequencing		5'-TCAGaCTCT-3'	
Product size (bp)		165	
Allele		A/G	

TaqMan		
**Primers**	**Sequence**	**Modification**
	**N485I**	
Forward	CATGCGATACTGGTCAAACTTTGC	
Reverse	GCCATTCGGGAAATTCGCTACTA	
reporter N485	CAAACCCCAACACGGC	VIC
Reporter 485I	CAAACCCCATCACGGC	FAM
	**A50T**	
Forward	ACGGCATCACGAACTCAACAA	
Reverse	GGCACCGGACAGCTGAT	
reporter A50	CCGATGGTGGCCCCA	VIC
Reporter 50T	CGATGGCGGCCCCA	FAM
Amplification Ex4-7 (967bp)	ACGCTCGGTATATGCGAGTT	TGACACTAGCAGCACAACCA

Green is *EcoRI*, Purple is *NdeI* and Red is *XbaI* site
