# Supplementary material for: The P450 CYP6Z1 confers carbamate/pyrethroid cross‐resistance in a major African malaria vector beside a novel carbamate‐insensitive N485I acetylcholinesterase‐1 mutation
Source: Mol Ecol. 2016 Jun 15;25(14):3436–52. doi: 10.1111/mec.13673 (PMC4950264; doi:10.1111/mec.13673)
Supplement: Supplementary file 3 — Appendix S1 Supplementary methods and results. [file MEC-25-3436-s003.docx]

**Appendix S1** Supplementary methods and results.
