# Supplementary material for: The P450 CYP6Z1 confers carbamate/pyrethroid cross‐resistance in a major African malaria vector beside a novel carbamate‐insensitive N485I acetylcholinesterase‐1 mutation
Source: Mol Ecol. 2016 Jun 15;25(14):3436–52. doi: 10.1111/mec.13673 (PMC4950264; doi:10.1111/mec.13673)
Supplement: Supplementary file 4 [file MEC-25-3436-s004.docx]

**Methods**

**Microarray:** The quantity and quality of the extracted RNA were assessed using a NanoDrop ND1000 spectrophotometer (Thermo Fisher, Waltham, MA USA) and Bioanalyzer (Agilent, Santa Clara, CA, USA), respectively. Complementary RNA (cRNA), labeled with cy3 or cy5 dye was amplified from each sample using the Agilent Quick Amp Labeling Kit (two-color) following the manufacturer’s protocol. cRNA quantity and quality were assessed before labeling using the NanoDrop and Bioanalyzer. Labeled cRNAs were hybridized to the arrays for 17 h at 65°C according to the manufacturer’s protocol. Five hybridizations were performed for each comparison (R_b_-S; R_b_-C; C-S; R_p_-S and R_p_-C) by swapping the biological replicates. Microarray data were analyzed using Genespring GX 12.0 software. To identify differentially expressed genes, a cut-off of 2-fold-change (FC) and a statistical significance of P<0.01 with Benjamini-Hochberg correction for multiple testing was applied.

**Heterologous co-expression of candidate P450 genes with *An. gambiae* P450 reductase**

**Cloning of candidate P450s for expression in *E. coli*:** Briefly, a DNA fragment containing the coding sequence for the ompA signal peptide with a downstream alanine-proline linker and approximately the first 20 nucleotides of each gene was first amplified using the specific primers (OMPA+2F and the reverse primer: OMPA+2-CYP6Z1R; Table S1) to create a linker fragment. The *CYP6Z1* selected clone and the ompA+2 PCR linker fragment were used as templates in a fusion PCR to generate a full length cDNA with ompA+2 modification in the NH_2_ terminus as previously carried out (Riveron et al. 2013). The full-length sequence incorporating the ompA+2 leader was then ligated into a modified pCW-ori + vector plasmid, pB13 (Pritchard et al. 1998), via *EcoR*I and *XBa*I sites to produce pB13::ompA+2-*CYP6PZ1*. This construct was sequenced to confirm the absence of PCR errors and used for protein expression.

**Pyrethroid and carbamate metabolism assays**

0.2M Tris-HCl and NADPH-regeneration components (1mM glucose-6-phosphate, 0.25mM MgCl_2_, 0.1mM NADP^+^ and 1U/mL glucose-6-phosphate dehydrogenase) were added to the bottom of 1.5ml tube chilled on ice. 45pmol of membrane expressing appropriate P450 and reductase, and cytochrome b_5_ were added to the side of the tube and pre-incubated for 5 minutes at 30^o^C with shaking at 1200 rpm to activate the membrane. 20µM of test insecticide was then added into the final volume of 0.2ml (less than 2.5% v/v methanol) and reaction started by vortexing at 1200 rpm and 30^o^C for 1 hour. Reactions were quenched with 0.1ml ice-cold methanol and incubated for 5 more minutes at 1200rpm, to precipitate protein. Tubes were then centrifuged at 16400 rpm and 4^o^C for 15 minutes and 150µl of supernatant transferred into HPLC vials. All reactions were carried out in triplicates with experimental samples (+NADPH) and negative control (-NADPH) not containing NADP in the regeneration system. 100µL of sample was loaded into an isocratic mobile phase of Agilent 1260 Infinity with a flow rate of 1ml/min and peaks separated with a 250mm C18 column (Acclaim ^TM^ 120, Dionex) at 23^o^C for pyrethroids and 40^o^C for bendiocarb and propoxur. In a mobile phase of 65:35 v/v acetonitrile:water, bendiocarb eluted between 13-14 minutes and was detected at 205nm while propoxur was detected at 270nm with retention time established as ~6 minutes in a mobile phase of 60:40 v/v acetonitrile:water. Enzyme activity was calculated as the percentage depletion (the difference in the amount of insecticide(s) remaining in the +NADPH tubes compared with the –NADPH) and a paired t-test was used for statistical analysis.

**Fluorescent probes assay and kinetics**

Reaction was conducted at 37^o^C using the fluorescent spectrophotometer Infinity® M200 (TECAN). Resorufin, coumarin and fluorescein formation were monitored at excitation/emission wavelengths of 544/590nm, 410/535nm and 485/530nm respectively. Rate of fluorescent product formation was determined as relative fluorescence per minute by linear regression of measurement between 2 and 8 minutes after addition of NADPH generating components. Results were analyzed with associated Magellan^TM^ v6.2 Wizard.

For kinetics, 0 to 2µM DEF was assayed with 3.33pmol membranes in a total volume of 250µl/well. The protocol was as outlined above, only that the substrate concentration varies and incubation was done under conditions shown to be linear with respect to time, substrate and enzyme concentrations. Steady-state kinetic parameters were obtained by measuring the rate of reaction for 10 minutes while varying the substrate concentration from 0 to 2µM. K_M_ and Vmax were established from the plot of substrate concentrations against the initial velocities through a non-linear regression by fitting the data to the Michaelis-Menten equation using GraphPad Prism 6.03 (GraphPad Software Inc., La Jolla, CA, USA). Catalytic constants and efficiencies were determined from the steady-state parameters.

**Fluorometric inhibition assay**: In a total volume of 220µl containing test insecticides (inhibitors) or miconazole serially diluted into 8-fold concentrations (25-0.011µM) and buffered with 50mM potassium phosphate buffer (at pH 7.4 with 5mM MgCl_2_), 3.33pmol membrane and ~0.13µM DEF substrate was added to give 225µl. After pre-incubation for 5 minutes at 37^o^C, reaction was started by addition of 25µl of regeneration component. The regeneration component comprises 7.8mg glucose-6-phosphate (G6P), 0.25mM MgCl_2_, 1.7mg NADP, 6U/mL glucose-6-phosphate dehydrogenase and 2% w/v NaHCO_3_. Fluorescence was monitored for 21 cycles at interval of 1 minute with shaking at every step.

**Sequencing of full-length *ace-1* and *ace-2*:** Both genes were amplified using the Phusion High-Fidelity DNA Polymerase (Fermentas) in a 25µl reaction containing 10pmol of each primers, 30ng of cDNA as template, 1X Phusion® HF Buffer buffer, 0.2mM dNTPs, 1.5mM MgCl_2_, 1U Phusion Polymerase. The PCR conditions were as follows: 1 cycle at 95°C for 5 min; 35 cycles of 94°C for 20s, 60°C for 30s and elongation at 72°C for 90s; and 1 cycle at 72°C for 5 min. The primers used are listed in Table S1. The PCR products were purified using the QIaquick PCR Purification Kit (Qiagen, Valencia, CA) and cloned into the pJET1.2/blunt cloning vector using the CloneJET^TM^ PCR Cloning Kit (Fermentas). Positive clones were purified by QIAprep® Miniprep (Qiagen, Valencia, CA) and sequenced on both strands. Polymorphic positions were detected through manual analysis of sequence traces using BioEdit and as sequence differences in multiple alignments using ClustalW (Thompson et al. 1994).

**Results**

**Transcription profiling of bendiocarb resistance using microarray**

To detect the genes associated with carbamate resistance in populations of *An. funestus*, the 4x44k (A-MEXP-2245)(Riveron et al. 2013) and the 8x60k (A-MEXP-2374)(Riveron et al. 2014) Agilent microarray chips (Agilent) were used to perform a genome-wide transcription profiling. Labelled complementary RNA (cRNA) were generated from 3 biological replicates for the following samples: i) bendiocarb resistant (R_b_) (mosquitoes alive after 1h exposure to 0.1% bendiocarb), ii) control (C) (mosquitoes unexposed to insecticide thus representative of the wild type population), and iii) susceptible (S) (unexposed mosquitoes from the fully susceptible laboratory strain FANG). These cRNA were reciprocally hybridized against each other in a triangle design with three types of comparisons; R_b_-S for bendiocarb Resistant vs Susceptible, R_b_-C for bendiocarb Resistant vs Control for bendiocarb resistance profiling and the C-S hybridization for Control vs Susceptible. Candidate genes are identified in this experimental design while taking into account the difference in genetic background between strains. Additionally, these bendiocarb hybridization profiles were compared to those from permethrin resistant mosquitoes (Riveron et al. 2013) to detect genes associated with cross-resistance. Additional details are given in File S1: Results.

The R-S comparison exhibited a total of 4088 differentially expressed at P<0.01; of which 2229 probes were up-regulated in the bendiocarb resistant mosquitoes while 1859 probes were down-regulated in comparison to the susceptible FANG strain (S) (File S3: Figure S1A). The R-C comparison had 2366 probes differentially expressed at P<0.01 of which 1180 were up-regulated and 1186 were down-regulated respectively. The C-S had 3295 probes at P<0.01 of which 2237 were up-regulated and 1058 were down-regulated respectively. The R-C, C-S and R-S all shared 504 probes in common, of which 81 probes were up-regulated and 423 were down-regulated. The R-S shared 1391 probes with the C-S, and also shared 334 probes with R-C. The R-C shared 342 probes with the C-S.

**Transcriptional analysis:**

**Genes expressed in R-C and/or R-S:** Several probes belonging to salivary gland protein genes were equally over-expressed, notably the D7-related 1 protein gene which was highly over-expressed (FC=20 in R-C). Other genes families detected are commonly associated with insecticide resistance such as peroxidases, heat shock proteins and proteases such as Trypsin. A set of probes belonging to immune response genes were also over-expressed including the TEP1 gene and several CLIP genes. A set of three probes belonging to trypsin is over-expressed in both R-C and R-S but are all highly down-regulated in the C-S comparison.

**Genes over-expressed only in R-C:** The list of probes up-regulated in R-C only is dominated by P450 genes such as CYP6M4, CYP325D1 and CYP4H18. This list also includes other genes such as the glutathione-S transferase GSTO1, an aldehyde oxidase, an arginino-succinate lyase, several peroxidases, odorant receptor genes, salivary proteins or heat shock proteins.

**Search for potential resistance mutations by sequencing of full-length *ace-1*:** The full-length cDNA of the acetylcholinesterase gene was successfully amplified, cloned and sequenced from bendiocarb resistant *An. funestus* samples from Malawi, Mozambique and Benin with a total size of 2208 bp corresponding to 736 amino acids. However, an alternative splicing was observed at exon 2 with a 8 amino acid sequence (DAFFTPYI) (highly conserved between *An. funestus*, *An. gambiae* and *Culex quinquefasciatus*) present at the beginning of this exon for some clones. *An. funestus* *ace-1* shares 96% similarity with that of *An. gambiae* (AGAP001356-RA), 91% with that of *Aedes aegypti* (AAEL000511-RA), 81.9% with that of *Culex quinquefasciatus*.

**Prediction of structural impact of N485I through homology modelling of *ace-1***

The two generated models with the template 2C58 revealed a conservative folding with typical canonical catalytic triad residues Ser^358^ (Ser^200^), Glu^484^ (Glu^327^) and His^598^ (His^440^) of the esteratic subsite (number in parenthesis indicates *Tc*AChE nomenclature) (Quinn 1987; Sussman et al. 1991) occupying identical positions with the template. Overall 3D folding was found to be well-conserved with other residues (regions) including the Trp^84^, Glu^199^ and Tyr/Phe^330^ of the choline binding ‘anionic’ site, the Gly^118^, Gly^119^ and Ala^201^ of the oxyanion hole, as well as the rest of the well-conserved aromatic residues lining the active site gorge were all predicted to be in the same position, as in the template (Weill et al. 2004; Dvir et al. 2010). While most of the mutations that impact on insects acetylcholinesterase activity were recorded to map to the location within or adjacent to the active site gorge (Fournier 2005), in contrast the *An. funestus ace1* Asn^485^Ile mutation mapped to the external loop joining the α^2^_8,9_ with β9 (Koellner et al. 2000), considerable distance away from the catalytic site (Figure 5D). In addition the only difference observed between the wild type and mutant model was in this external loop distorted inward in the mutant model by 3.8Å compared with the wild type model and the template, 2C58. Residues from this region have been described as outliers in the several resolved structures of *Tc*AChE. For example, Pro^485^ of *Tc*AChE was described as an outlier with real space R-value Z-score of 3.4 (Weik et al. 2000). The residue was also described not to be seen in the crystal of *T. californica* 1ACE resolved at 2.8Å (Axelsen et al. 1994). Residues 1-3 and the exposed loop (485-489) of crystal structure of *Tc*AChE were also reported not to be seen in an electron density map probably because they are disordered (Sussman et al. 1991).

Axelsen PH, Harel M, Silman I, Sussman JL. 1994. Structure and dynamics of the active site gorge of acetylcholinesterase: synergistic use of molecular dynamics simulation and X-ray crystallography. *Protein science : a publication of the Protein Society* **3**: 188-197.

Dvir H, Silman I, Harel M, Rosenberry TL, Sussman JL. 2010. Acetylcholinesterase: from 3D structure to function. *Chemico-biological interactions* **187**: 10-22.

Fournier D. 2005. Mutations of acetylcholinesterase which confer insecticide resistance in insect populations. *Chem Biol Interact* **157-158**: 257-261.

Koellner G, Kryger G, Millard CB, Silman I, Sussman JL, Steiner T. 2000. Active-site gorge and buried water molecules in crystal structures of acetylcholinesterase from Torpedo californica. *Journal of molecular biology* **296**: 713-735.

Pritchard MP, Glancey MJ, Blake JA, Gilham DE, Burchell B, Wolf CR, Friedberg T. 1998. Functional co-expression of CYP2D6 and human NADPH-cytochrome P450 reductase in Escherichia coli. *Pharmacogenetics* **8**: 33-42.

Quinn DM. 1987. Acetylcholinesterase: enzyme structure, reaction dynamics, and virtual transition states. *Chemical Reviews* **87**: 955-979.

Riveron JM, Ibrahim SS, Chanda E, Mzilahowa T, Cuamba N, Irving H, Barnes KG, Ndula M, Wondji CS. 2014. The highly polymorphic CYP6M7 cytochrome P450 gene partners with the directionally selected CYP6P9a and CYP6P9b genes to expand the pyrethroid resistance front in the malaria vector Anopheles funestus in Africa. *BMC Genomics* **15**: 817.

Riveron JM, Irving H, Ndula M, Barnes KG, Ibrahim SS, Paine MJ, Wondji CS. 2013. Directionally selected cytochrome P450 alleles are driving the spread of pyrethroid resistance in the major malaria vector Anopheles funestus. *Proc Natl Acad Sci U S A* **110**: 252-257.

Sussman JL, Harel M, Frolow F, Oefner C, Goldman A, Toker L, Silman I. 1991. Atomic structure of acetylcholinesterase from Torpedo californica: a prototypic acetylcholine-binding protein. *Science* **253**: 872-879.

Thompson JD, Higgins DG, Gibson TJ. 1994. CLUSTAL W: improving the sensitivity of progressive multiple sequence alignment through sequence weighting, position-specific gap penalties and weight matrix choice. *Nucleic Acids Res* **22**: 4673-4680.

Weik M, Ravelli RB, Kryger G, McSweeney S, Raves ML, Harel M, Gros P, Silman I, Kroon J, Sussman JL. 2000. Specific chemical and structural damage to proteins produced by synchrotron radiation. *Proceedings of the National Academy of Sciences of the United States of America* **97**: 623-628.

Weill M, Malcolm C, Chandre F, Mogensen K, Berthomieu A, Marquine M, Raymond M. 2004. The unique mutation in ace-1 giving high insecticide resistance is easily detectable in mosquito vectors. *Insect Mol Biol* **13**: 1-7.
